# Early access to antiretroviral therapy versus standard of care among HIV‐positive participants in Eswatini in the public health sector: the MaxART stepped‐wedge randomized controlled trial

**DOI:** 10.1002/jia2.25610

**Published:** 2020-09-19

**Authors:** Shaukat Khan, Donna Spiegelman, Fiona Walsh, Sikhatele Mazibuko, Munyaradzi Pasipamire, Boyang Chai, Ria Reis, Khudzie Mlambo, Wim Delva, Gavin Khumalo, Mandisa Zwane, Yvette Fleming, Emma Mafara, Anita Hettema, Charlotte Lejeune, Ariel Chao, Till Bärnighausen, Velephi Okello

**Affiliations:** ^1^ Clinton Health Access Initiative (CHAI) Mbabane Swaziland; ^2^ Center on Methods for Implementation and Prevention Science and Department of Biostatistics Yale School of Public Health New Haven CT USA; ^3^ Departments of Epidemiology Biostatistics, Nutrition and Global Health Harvard T.H Chan School of Public Health Boston MA USA; ^4^ Clinton Health Access Initiative (CHAI) Boston MA USA; ^5^ Eswatini National ART program (SNAP) Ministry of Health Mbabane Swaziland; ^6^ Department of Nutrition Harvard T.H Chan School of Public Health Boston MA USA; ^7^ Leiden University Medical Center Leiden University Leiden the Netherlands; ^8^ Amsterdam Institute for Social Science University of Amsterdam Amsterdam the Netherlands; ^9^ Children's Institute University of Cape Town Cape Town South Africa; ^10^ The South African Department of Science and Technology ‐ National Research Foundation (DST‐NRF) Centre of Excellence in Epidemiological Modelling and Analysis (SACEMA) Stellenbosch University Stellenbosch South Africa; ^11^ Center for Statistics Hasselt University Diepenbeek Belgium; ^12^ International Centre for Reproductive Health Ghent University Gent Belgium; ^13^ KU Leuven Rega Institute for Medical Research Leuven Belgium; ^14^ Department of Global Health Faculty of Medicine and Health Sciences Stellenbosch University Stellenbosch South Africa; ^15^ Eswatini National Network of People Living with HIV (SWANNEPHA) Mbabane Swaziland; ^16^ SAfAIDS Manzini Swaziland; ^17^ aidsfonds Amsterdam the Netherlands; ^18^ Heidelberg Institute of Public Health University of Heidelberg Heidelberg Germany; ^19^ Department of Global Health and Population Harvard T.H Chan School of Public Health Boston MA USA; ^20^ Directorate Office Ministry of Health Mbabane Swaziland

**Keywords:** universal treatment, HIV retention, viral suppression, Eswatini, Sub‐Saharan Africa, antiretroviral therapy

## Abstract

**Introduction:**

The WHO recommends antiretroviral treatment (ART) for all HIV‐positive patients regardless of CD4 count or disease stage, referred to as “Early Access to ART for All” (EAAA). The health systems effects of EAAA implementation are unknown. This trial was implemented in a government‐managed public health system with the aim to examine the “real world” impact of EAAA on care retention and viral suppression.

**Methods:**

In this stepped‐wedge randomized controlled trial, 14 public sector health facilities in Eswatini were paired and randomly assigned to stepwise transition from standard of care (SoC) to EAAA. ART‐naïve participants ≥18 years who were not pregnant or breastfeeding were eligible for enrolment. We used Cox proportional hazard models with censoring at clinic transition to estimate the effects of EAAA on retention in care and retention and viral suppression combined.

**Results:**

Between September 2014 and August 2017, 3405 participants were enrolled. In SoC and EAAA respectively, 12‐month HIV care retention rates were 80% (95% CI: 77 to 83) and 86% (95% CI: 83 to 88). The 12‐month combined retention and viral suppression endpoint rates were 44% (95% CI: 40 to 48) under SoC compared to 80% (95% CI: 77 to 83) under EAAA. EAAA increased both retention (HR: 1·60, 95% CI: 1·15 to 2·21, *p* = 0.005) and retention and viral suppression combined (HR: 4.88, 95% CI: 2.96 to 8.05, *p* < 0.001). We also identified significant gaps in current health systems ability to provide viral load (VL) monitoring with 80% participants in SoC and 66% in EAAA having a missing VL at last contact.

**Conclusions:**

The observed improvement in retention in care and on the combined retention and viral suppression provides an important co‐benefit of EAAA to HIV‐positive adults themselves, at least in the short term. Our results from this “real world” health systems trial strongly support EAAA for Eswatini and countries with similar HIV epidemics and health systems. VL monitoring needs to be scaled up for appropriate care management.

## 1 Introduction

In 2018, nearly 38 million people were living with human immunodeficiency virus (HIV), of whom 20.64 million live in sub‐Saharan Africa. There has been a 56% decline in AIDS‐related deaths from the peak of 1.7 million in 2004 to 770,000 in 2018, likely due to the three‐fold increase in people receiving antiretroviral therapy (ART) since 2010 [1]. While there has been significant progress to scale up HIV testing and treatment, the incidence of HIV infection remains high, with an estimated 1.7 million newly infected people in 2018 [1].

The HPTN 052 trial was first to demonstrate that early antiretroviral (ART) initiation – referred to here as Early Assess to ART for All (EAAA) – prevents viral transmission to the uninfected partner in heterosexual HIV‐discordant couples [2]. To understand if the reported efficacy of EAAA would have similar effectiveness in preventing transmission in sub‐Saharan Africa, four large community‐based trials were launched between 2011 and 2013 [3, 4, 5, 6, 7, 8]. These trials demonstrated that there are significant implementation barriers to EAAA for reducing HIV incidence in high‐prevalence resource‐limited settings.

In 2014, the Eswatini Ministry of Health launched the MaxART stepped‐wedge randomized controlled trial to implement EAAA through the government‐managed health system. Eswatini has one of the highest HIV prevalence rates in the world, 27%, and an estimated annual incidence of 1.36% in adults [9]. While the Eswatini Ministry of Health had made substantial progress in addressing their HIV epidemic, there was interest in identifying an effective, sustainable approach to reduce the infection rate. Without this reduction, the number of people needing HIV treatment in Eswatini will likely expand along with the treatment costs.

In September 2015, the World Health Organization (WHO) recommended ART irrespective of CD4 count (EAAA) [10]. As high‐prevalence countries adopt these new guidelines, the MaxART trial offers evidence about the “real‐world” impact on retention and viral suppression under an EAAA approach. This trial is registered at clinicaltrials.gov (NCT02909218).

## 2 Methods

This trial was designed and conducted by the MaxART Consortium – led by the Eswatini Ministry of Health – from September 2014 to August 2017. The trial methods and power calculations have been reported elsewhere [11]. Briefly, we implemented a clinic‐based stepped‐wedge design in which the participating 14 clinics were randomly assigned in pairs, matched by catchment size and geographic proximity, to shift from standard of care (SoC) to EAAA at pre‐specified dates. After four months of baseline data collection in all clinics, one pair of clinics transitioned at each four‐month step. The last pair was transitioned on 1 October 2016 instead of 1 January 2017, because on the former date, Eswatini changed its SoC from CD4 ≤ 500 to EAAA. This change was approved by the trial’s Data Safety Monitoring Board, Advisory Board, Primary Research Team and the Eswatini Ministry of Health. The health workers, trial participants and trial staff were blinded to the timing of the clinic transition to minimize bias.

### 2.1 Participants

ART‐naïve, non‐pregnant or breastfeeding HIV‐positive participants, 18 years of age or older, with no mental illness were eligible for the trial. Individuals who had already been initiated on ART before the start of the trial were excluded. All eligible participants were enrolled.

### 2.2 Standard of care

Under SoC, participants meeting SoC ART eligibility criteria were referred for ART initiation, while those who did not were enrolled in pre‐ART care. Pre‐ART participants received follow‐up appointments to re‐assess ART eligibility and screen for tuberculosis symptoms in three‐month intervals. Over the course of the trial, changes to the national SoC for ART eligibility were incorporated into the trial implementation. Prior to 1 December 2015, the national ART initiation threshold was CD4 ≤ 350 and/or WHO Stage 3 or 4. On 1 December 2015, the threshold changed to CD4 ≤ 500 and/or WHO Stage 3 or 4, and on 1 October 2016, Eswatini adopted EAAA as SoC.

### 2.3 Early access to ART for all

Under EAAA, all HIV‐positive trial participants were eligible for ART initiation irrespective of CD4 count or WHO stage. Prior to the intervention, there were community sensitization events with traditional leaders in the surrounding communities, and health talks and posters about EAAA at the participating clinics. Clinical mentors were also positioned at the participating facilities to support the nursing staff with the introduction of EAAA.

### 2.4 Measurements

The primary endpoints – retention in HIV care and viral suppression six months or more after ART initiation among those retained – were chosen because they are proxy indicators for long‐term HIV‐related health outcomes, mortality and transmission [12, 13]. We also present the effect of EAAA on a combined endpoint of retention and viral suppression. The results for several secondary endpoints (ART initiation, drug resistance, HIV diseases progression, cost per patient per year) for this trial are in preparation or under review at this time. The results for patient satisfaction, adherence and mortality have been published elsewhere [14, 15, 16].

The operational definitions of the primary endpoints, retention and viral suppression, were established according to the definitions outlined by the Eswatini Ministry of Health Integrated HIV Management Guidelines 2015 (Table 1). See Figures S1 and S2 for further details about these definitions. The combined endpoint was defined as the date when either non‐retention in care or elevated viral load (VL) occurred, and survival time was the minimum of the times from enrolment to any events qualifying for non‐retention or elevated VL. Unlike viral suppression, which considers the patients who survived six months or more beyond ART‐initiation, the combined endpoint is a fully randomized endpoint.

**Table 1:** Endpoint definitions

Primary endpoint	Definition	Additional considerations
Retention	Participant is classified as retained in HIV care if they are a) alive and b) have not stopped treatment, whereby either: [End of the Study Period]‐[Last Visit Date] < 90 days; or [Next Appointment Date] – [End of the Study Period]> (within 30 days) Time to non‐retention was the last date at which the first of the above defining events occurred.	If the next scheduled visit date is missing, then the next visit date will equal last visit date plus 120 days For participants who transferred out or stopped treatment, their last visit date was used as their transfer date or stopped treatment date
Viral suppression	Participants were excluded if: They were not retained for 180 days following ART initiation; or They were transferred out of the MaxART trial clinics by 180 days following ART initiation; or They were never initiated on ART; or The time between ART initiation and the study end date was <180 days Non‐excluded participants were classified as virally unsuppressed when one of the following was true: The first VL measurement was ≥1000 copies/mml; or They were lost to follow‐up by the end of the study or the clinic transition date; or They stopped treatment; or Their death was ART‐ or HIV‐related; or There was no available VL measurement	This endpoint assesses viral suppression conditional on ART‐initiation and retention to 180 days post ART initiation date Time to elevated VL 180 days post‐ART initiation was the date at which the first of these defining events occurred. As such, it is not a randomized endpoint When one or more VL measurements were available six months or more after ART initiation, we took the last available VL prior to censoring as the event‐defining value, assuming that if suppressed in the future, they were suppressed in the past
Combined retention and viral suppression	Defined as: Date when either non‐retention in care or elevated VL occurred, and Survival time was the minimum of the times from enrolment to any of the events qualifying for either non‐retention or elevated VL	While this combined endpoint was not one of the original primary endpoints registered for the trial, it offers important insights and was included in the final analysis The combined endpoint was analysed using an intent‐to‐treat approach

### 2.5 Data collection and management

We measured the endpoints using routine data extracted from the participants’ clinical records. We developed standard operating procedures for data management for transferring participant paper files to a trial‐specific electronic database. Nurse mentors regularly reviewed the participant files for accuracy and completeness, and provided mentorship to the clinic staff. The data manager and trial statistician generated monthly error reports for data clerks and nurse mentors to resolve in the clinics. In addition, the data manager conducted a quarterly quality assessment by reviewing 10% of paper records with electronic data entry.

### 2.6 Statistical analysis

We used Cox proportional hazard models for clustered data to estimate the effect size, confidence intervals and *p*‐values whenever between‐facility intra‐class correlations (ICCs) were greater than or equal to 0.01 [17, 18]. Given the small number of facilities, with very small ICCs, robust variances gave unrealistically small *p*‐values [19]. Because there were a small number of facilities randomized, residual confounding is possible. Thus, we conducted multivariable‐adjusted analyses, where we considered known and suspected baseline determinants of retention and viral suppression, and included those with *p* < 0.20 in the models [20]. Steptime was adjusted for in all analyses, to ensure that any background time trends related to quality of care or disease epidemiology did not lead to spurious differences between the two groups. Survival curves used the Breslow estimator and therefore needed to be estimated at specific covariate values in the multivariate Cox model from where the estimator was derived. The value of each covariate, including steptime, was set at the mean value of each person‐visit in the study population (Table 2). Stepwise restricted cubic splines were used to model continuous covariates, to reduces bias due to incorrect assumptions about linearity of the covariate‐outcome relationships [21]. To further adjust for any bias due to time trends not addressed by the categorical steptime variable, when calendar time as a linear or non‐linear effect was significant at *p* < 0.20 in any model, it was included in the multivariate model in addition to steptime. The missing indicator method was used to handle missing covariate data [22]. In sensitivity analyses for the viral suppression endpoint, Fine and Gray competing risk methods were used, treating mortality not HIV‐ or ART‐related as competing risks [23]. Confidence intervals and *p*‐values were not adjusted for multiplicity [24]. Effect modification was assessed by the robust score test, accounting for within‐facility correlation of the outcomes. Analysis of effect modification was conducted among the subset of participants not missing each covariate and adjusted for the baseline covariates given in Footnote 1 of Tables S1,S2,S3.

**Table 2:** Basic characteristics of MaxART participants at the time of trial enrolment

	Standard of Care (n = 2034)	EAAA (n = 1371)	All ART naïve (n = 3405)
Demographic characteristics			
Age at trial enrolment (year) median (q1, q3)	33 (28, 42)	33 (27, 40)	33 (27, 41)
Age group (years) n (%)			
18 to <20	40 (2%)	34 (3%)	74 (2%)
20 to <30	679 (33%)	472 (34%)	1151 (34%)
30 to <40	722 (36%)	500 (36%)	1222 (36%)
40 to <50	354 (17%)	230 (17%)	584 (17%)
50 to <60	156 (8%)	97 (7%)	253 (7%)
60+	83 (4%)	38 (3%)	121 (4%)
Sex n (%)			
Male	695 (34%)	603 (44%)	1298 (38%)
Female	1339 (66%)	768 (56%)	2107 (62%)
Marital status n (%)			
Married	1045 (52%)	634 (48%)	1679 (51%)
Divorced/widowed	127 (7%)	78 (6%)	205 (6%)
Single	825 (41%)	614 (46%)	1439 (43%)
Marital status missing, n (%)	37 (2%)	45 (3%)	82 (2%)
Education n (%)			
Illiterate/primary	589 (40%)	384 (38%)	973 (39%)
Secondary	438 (30%)	362 (36%)	800 (32%)
High school	401 (27%)	218 (22%)	619 (25%)
Tertiary	48 (3%)	37 (4%)	85 (4%)
Education missing, n (%)	558 (27%)	370 (27%)	928 (27%)
Clinical characteristics^a^			
BMI (kg/m^2^) n (%)			
<18.5	82 (4%)	81 (6%)	163 (5%)
18.5 to <25	971 (49%)	762 (57%)	1733 (52%)
25 to <30	529 (27%)	281 (21%)	810 (25%)
≥30	387 (20%)	212 (16%)	599 (18%)
BMI missing n (%)	65 (3%)	35 (3%)	100 (3%)
CD4 (cells/μL) n (%)			
<350	804 (44%)	632 (56%)	1436 (49%)
350 to 500	441 (24%)	224 (20%)	665 (22%)
>500	591 (32%)	266 (24%)	857 (29%)
CD4 missing n (%)	198 (10%)	249 (18%)	447 (13%)
WHO stage n (%)			
1	1074 (63%)	800 (63%)	1874 (63%)
2	357 (21%)	320 (25%)	677 (23%)
3 or 4	264 (16%)	146 (12%)	410 (14%)
WHO stage missing n (%)	339 (17%)	105 (8%)	444 (13%)
Screened for TB symptoms n (%)			
Positive	190 (10%)	100 (8%)	290 (9%)
Missing screening for TB symptoms n (%)	82 (4%)	66 (5%)	148 (4%)
Viral load (copies/ml) n (%)			
<1000	229 (12%)	120 (10%)	349 (11%)
1000 to <50‚000	781 (42%)	479 (40%)	1260 (41%)
50‚000 to <100‚000	213 (11%)	150 (13%)	363 (12%)
≥100‚000	661 (35%)	438 (37%)	1099 (36%)
Viral load missing n (%)	150 (7%)	184 (13%)	334 (10%)
Time between positive HIV test to trial enrolment (years) n (%)			
≤1	1189 (59%)	1076 (79%)	2265 (67%)
1 to ≤3	421 (21%)	134 (10%)	555 (16%)
>3	413 (20%)	144 (11%)	557 (17%)
Time from trial enrolment to ART initiation (months), median (q1, q3)	11 (5, 16)	0 (0, 0)	7 (0, 88)
Access to HIV Treatment supporter n (%)			
Yes	1980 (97%)	1339 (98%)	3319 (97%)
Clinic characteristics			
Level of clinic n (%)			
Hospital	449 (22%)	349 (26%)	798 (23%)
Clinic with maternity ward	317 (16%)	181 (13%)	498 (15%)
Clinic without maternity ward	1268 (62%)	841 (61%)	2109 (62%)
Clinic volume by ART visits at baseline^b^ n (%)			
< Median (400 visits)	915 (45%)	385 (28%)	1300 (38%)
≥ Median (400 visits)	1119 (55%)	986 (72%)	2105 (62%)

^a^ Values within 90 days of enrolment and before ART initiation

^b^ ART participant visits received during the first quarter into trial. Median number of visits during this period is 400.

The combined endpoint is a means of considering the impact of the intervention on viral suppression with less need for assumptions about those missing VLs. It has been reported that 91.9% of those who report being on ART are virally suppressed [25]. Thus, in sensitivity analysis, we varied the unsuppression rate among those missing VLs from 5% to 10% to 20%, randomly assigning those who experience the combined endpoint through missing VL to being considered unsuppressed at the assumed percentage.

### 2.7 Ethical approval

The trial was approved by the Eswatini National Health Research Review Board on 17 July 2014 (MH/599C/FWA 000 15 267).

## 3 Results

**Figure 1:**
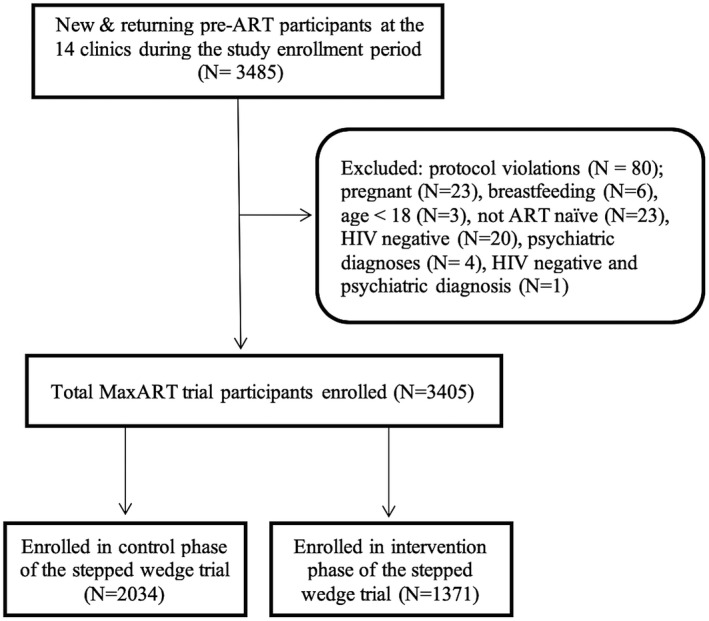
Flow diagram of participants enrolled during the control and intervention phases MaxART

**Figure 2:**
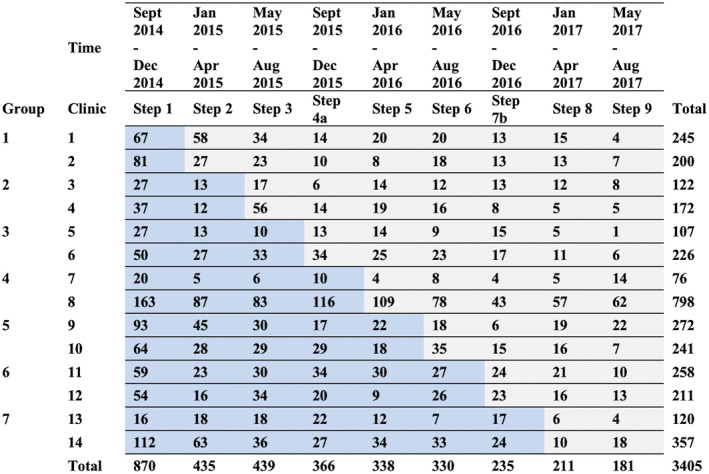
Number of enrolled participants by clinic pairs and steps. *Eswatini national guidelines for ART initiation changed from a CD4 count threshold of ≤ 350 cells/μL to ≤ 500 cells/μL on December 1, 2016. **Eswatini’s national ART guidelines for ART initiation changed a CD4 count threshold of ≤ 500 cells/μL or less to a universal test and treat approach on 1 October 2016

The trial was conducted between September 2014 and August 2017, during which 3485 participants were enrolled. Following *a priori* exclusion criteria, 80 (2%) participants were excluded, leaving 3405 eligible participants, of whom 2034 (60%) enrolled during the SoC phase of their clinic and 1371 (40%) under EAAA (Figure 1). From the distribution of enrolment by clinic and step, we observed a larger sample size in the earlier steps because existing pre‐ART participants were enrolled into the trial, whereas at later steps of the trial, only participants who were newly diagnosed or who had previously disengaged from pre‐ART care remained available for enrolment (Figure 2). Consequently, there were more participants in SoC than EAAA.

Demographic characteristics did not vary appreciably between the two study arms, with the exception of sex (34% SoC and 44% EAAA of the enrolled participants were male). Most clinical characteristics did vary between the study arms. Notably, 79% of participants enrolled within a year of HIV diagnosis in EAAA, compared to 49% in SoC, and 56% enrolled with baseline CD4 ≤ 350 in EAAA compared to 44% in SoC (Table 2).

**Figure 3:**
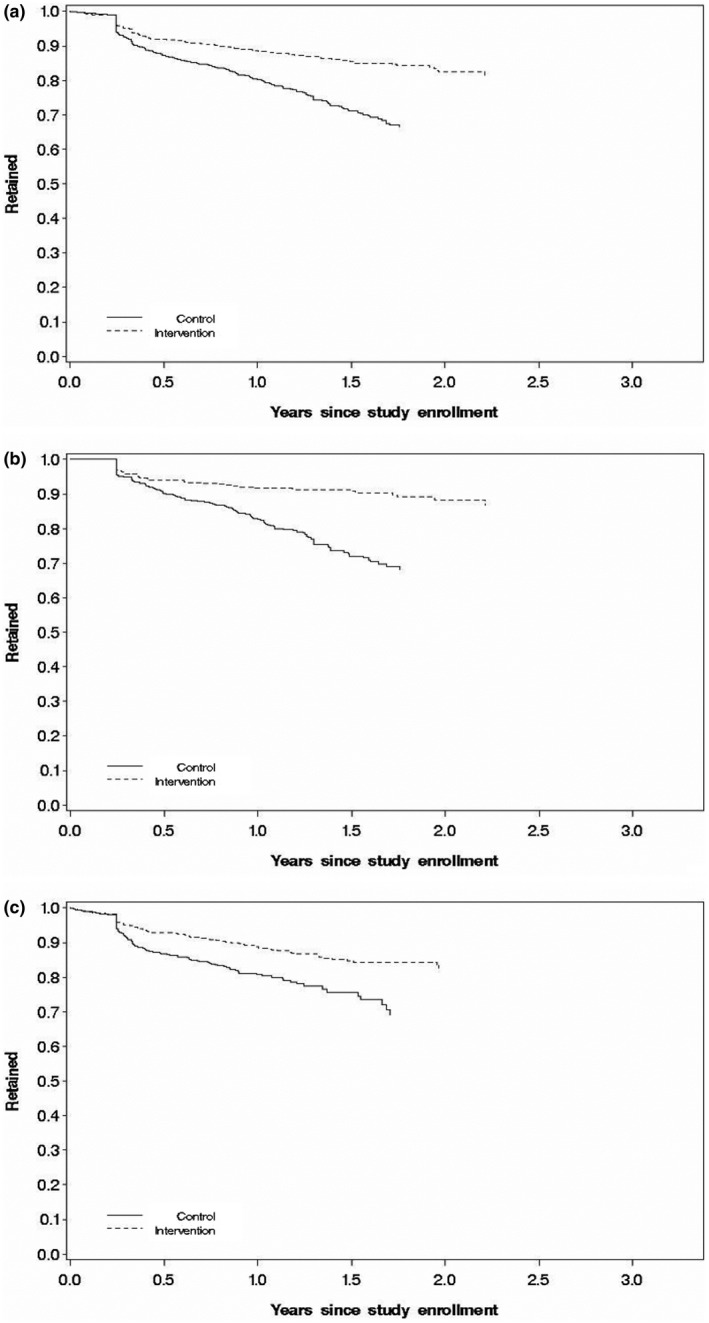
Kaplan–Meier curves for retention. **(a)** all participants, **(b)** above SoC and **(c)** under SoC. Graphs reflect the cumulative incidence averaged over covariates. Full multivariable model adjusted for steptime, age (18‐<20 years old, 20‐<30 years old, 30‐<40 years old, 40‐<50 years old,5 0‐<60 year old, 60 + years old), sex, marital status (Married, Divorced/Widowed, Single), education (Illiterate/Primary, Secondary, High School, Tertiary), CD4 (<350, 350 to 500, >500), WHO stage (stage 1, stage 2, stage 3 or 4), BMI (<18.5, 18.5‐<25, 25 to <30, ≥30), screened for TB symptoms (yes, no), viral load (<5‚000, 5‚000 to 30‚000, >30‚000), access to HIV treatment supporter (yes, no), level of clinic (Hospital, Clinic with maternity, Clinic without maternity), time from HIV tested positive to enrolment (≤1 year, 1‐ ≤3 years, >3 years), clinic volume (Low: < median, High: ≥ median). Missing data were treated as a separate group for each of the covariates in the models. All adjusted variables were derived at study enrolment. Marginal model was used to adjust for clustering effect among facilities. SoC‐ineligible also included an additional variable for study enrolment date (continuous) in the model

Table 3 provides the steptime‐adjusted incidence rates and hazard ratios for the three endpoints. In SoC and EAAA, respectively, the 12‐month retention rates were 80% (95% confidence interval (CI): 77 to 83) and 86% (95% CI: 83 to 88) (Figure 3). Under EAAA compared to SoC, a 60% significant improvement in retention was observed (HR: 1.60, 95% CI: 1.15 to 2.21, *p* = 0.005) (Table 3). Although VL coverage was low in both the SoC and EAAA arms with 80% and 66% of participants not receiving a VL between 180 days post‐ART initiation and end of follow‐up, significant improvements of increased viral suppression were also observed with EAAA compared to SoC (HR: 14.51, 95% CI: 7.31 to 28.79, *p* < 0.0001). In SoC and EAAA, respectively, the viral suppression rates, at 12 months post‐ART initiation among those retained, were 4% (95% CI: 2 to 7) and 79% (95% CI: 75 to 83) (Figure S3). Lastly, estimates for the combined endpoint can be interpreted as an assessment of the impact of the intervention on the third 90 of the HIV care cascade [26, 27]. At one year, the cumulative incidence of retention and viral suppression was 44% (95% CI: 40 to 48) under SoC compared to 80% (95% CI: 77 to 83) under EAAA, and at two years, it was 5% (95% CI: 3 to 9) under SoC compared to 60% (95% CI: 56 to 65) under EAAA (Figure S4). EAAA led to a large significant increase in the combined endpoint (HR: 4.88, 95% CI: 2.96 to 8.05, *p* < 0.0001) (Table 3).

**Table 3:** The effect of EAAA on retention and viral suppression six months post ART initiation

Population	Control			Intervention			Steptime adjusted		Multivariable adjusted		
	Number of Failures	Follow‐up time (person‐years)	Rate per person‐year	Number of Failures	Follow‐up time (person‐years)	Rate per person‐year	HR (95%CI)^a^	*p*‐value^b^	HR (95%CI)^a^	*p*‐value^b^	
Retention											
All enrolled participants	316	1665.7	0.19	215	1364.6	0.16	1.60 (1.15 to 2.21)	0.005	1.94 (1.33 to 2.82)^c^	0.0006	
SoC‐ineligible	150	851.7	0.18	48	399.6	0.12	1.89 (1.21 to 2.96)	0.005	2.75 (1.69 to 4.50)^d^	<0.0001	
SoC‐eligible	138	661.9	0.21	117	839.7	0.14	1.79 (1.20 to 2.66)	0.004	1.84 (1.28 to 2.65)^c^	0.0009	
Viral suppression six months post ART initiation											
All enrolled participants	352	386.9	0.91	308	691.1	0.45	14.51 (7.31 to 28.79)	<0.0001	22.08 (7.91 to 61.59)^e^	<0.0001	
SoC‐ineligible	114	121.9	0.94	65	232.7	0.28	13.79 (6.11 to 31.11)	<0.0001	19.91 (6.14 to 64.61)^e^	<0.0001	
SoC‐eligible	225	249.5	0.90	205	428.4	0.48	16.18 (8.31 to 31.51)	<0.0001	30.86 (8.89 to 107.20)^f^	<0.0001	
Combined retention and viral suppression											
All enrolled participants	723	1649.8	0.44	516	1332.2	0.39	4.88 (2.96 to 8.05)		6.90 (3.11 to 15.31)^g^		
SoC‐ineligible	275	847.8	0.32	110	395.2	0.28	4.17 (2.38 to 7.32)		6.22 (2.71 to 14.30)^c^		
SoC‐eligible	402	650.0	0.62	308	813.7	0.38	6.51 (4.07 to 10.42)		9.25 (4.53 to 18.91)^h^		

^a^ HR compared to standard of care rates

^b^ Not adjusted for multiplicity

^c^ adjusted for steptime, age at study enrolment (18 to <20 years old, 20 to <30 years old, 30 to <40 years old, 40 to <50 years old, 50 to <60 years old, 60+ years old), sex, marital status (married, divorced/widowed, single), education (illiterate/primary, secondary, high school, tertiary), CD4 counts (<350, 350 to 500, >500), WHO stage (stage 1, stage 2, stage 3 or 4), BMI (<18.5, 18.5 to <25, 25 to <30, ≥30), screened for TB symptoms (yes, no), viral load (<5000, 5000 to 30‚000, >30‚000), treatment support (yes, no), level of clinic (Hospital, Clinic with maternity, Clinic without maternity), time from HIV tested positive to enrolment (≤1 year, 1 to ≤3 years, >3 years), clinic volume (Low: < median, High: ≥median

^d^ same as footnote 2, plus study enrolment date (continuous)

^e^ came as footnote 2, plus study enrolment date (2 knot restricted cubic splines). If CD4/WHO at 180 days post ART initiation was not available, use CD4/WHO at ART initiation if available, or CD4/WHO at study enrolment if available. For WHO stage at 180 days post ART initiation, which had too few missing to use the missing indicator method, the missings were assigned to the reference category.

^f^ same as footnote 4

^g^ same as footnote 2, plus study enrolment date (2 knots stepwise restricted cubic spline)

^h^ same as footnote 2, plus study enrolment date (3 knots stepwise restricted cubic spline).

In addition, we carried out subgroup analyses in participants eligible for ART at the time of enrolment under the current SoC (“SoC eligible”), and in participants eligible only under EAAA (“SoC ineligible”). There was strong evidence of similar rates of improvement in retention, viral suppression at six months or more after ART initiation, and the combined endpoint for both subpopulations (Table 3 and Figure S5). The estimated effect sizes and significance levels for both retention and viral suppression increased after multivariable adjustment (Table 3).

In our analysis, 74% of the unsuppressed VLs were due to the lack of any measurements six months or more following ART initiation. Among those lacking measurements, 50% of those participants were missing VL due to no clinic visits during this time, with the other 50% had missing VL despite having had a least one clinical visit in this time window. In an attempt to separate out the effects of EAAA itself from the health system capacity to conduct adequate VL monitoring, we conducted several sensitivity analyses, with multivariable‐adjusted results as follows: 1) Including only participants with VL measured after baseline, the effect of EAAA on viral suppression and ability to provide VL measurements six months or more after ART initiation among those retained was 3.60 (95% CI: 2.13 to 6.09) times higher among those under EAAA compared to SoC. 2) Considering the most extreme scenario for those missing VLs – that participants enrolled under EAAA were considered unsuppressed, and participants enrolled under SoC were considered suppressed – we found that EAAA participants were 2.09 (95% CI: 1.26 to 3.46) times as likely to be suppressed by end of follow‐up compared to SoC participants. 3) Considering that those with missing VLs six months or more following ART initiation were treated as suppressed at all clinic visits, assuming that even if there were no VLs recorded, participants were appropriately treated at their visits, results were substantially attenuated in this analysis: the steptime‐adjusted HRs (95% CI, *p*‐value) were 1.89 (1.13 to 3.17, 0.01) compared to 14.51 (7.31 to 28.79, <0.001) in the original approach. These two results can be considered an upper and lower bound on the effectiveness of EAAA on viral suppression six months post ART initiation among those retained. Since some participants attending clinic without VLs were likely unsuppressed, if all VLs were available, the findings would likely be somewhere in between these two bounds. Of course, missed clinic visits leads to non‐adherence, since the provision of medications was matched to the clinic visit schedule; thus, it is reasonable to treat missing clinic visits as unsuppressed VLs. Clearly, the strong effect of EAAA on viral suppression was both a function of the EAAA itself as well as the enhanced health system capabilities. Competing risk analysis for this endpoint produced HRs materially unchanged from the primary analysis (data not shown).

Furthermore, in sensitivity analysis for the combined endpoint, as we varied the unsuppression rate among those with missing VL from 5% to 20%, the benefit of the combined endpoint as HRs increased from 1.84 (95% CI: 1.23 to 2.77) to 2.20 (95% CI: 1.42 to 3.41), demonstrating that even with very high suppression rates among those with missing VLs, there was still an increased rate of retention and/or viral suppression associated with EAAA.

Finally, after consideration of multiple comparisons, there were only three significant instances of effect modification on any of the three endpoints by covariates (Figures S6,S7,S8 and Tables S1,S2,S3). Viral suppression and the combined endpoint varied significantly by facility level, with EAAA having the strongest effect on these endpoints in hospitals, and the weakest in clinics with no maternity facilities. We did not assess effect modification by clinic for the viral suppression endpoint because some clinics had no participants available for assessment.

## 4 Discussion

This is the first randomized controlled trial to establish the impact of EAAA on the critical endpoints of ART retention and viral suppression in a “real‐world” public sector health system setting in sub‐Saharan Africa. Increased retention and viral suppression at six months or more after ART initiation was observed with EAAA compared to SoC, similar to previous observational data showing that those immediately eligible for ART had higher retention rates under existing national guidelines, than participants above the eligibility criteria [28, 29]. EAAA led to both a 60% increase in retention rates and – when considering documented viral load – a fivefold increase in retention and viral suppression combined. VL suppression can be considered a proxy measurement of health system efficiency, simultaneously assessing retention, the ability of the healthcare system to provide a VL result, and when available, to maintain suppression. When only those with at least one VL measurement post‐ART are included, the 6‐ and 12‐month suppression rates were 0.94 and 0.91 in the EAAA participants, and 0.82 and 0.72 among SoC participants (Figure S3). However, when we assume that those who died, were lost to follow‐up or were likely untreated due to not having VLs measured were all unsuppressed, as in the definition given in Figure S2, the six‐ and twelve‐month suppression rates were 0.90 and 0.79 in the EAAA participants, and 0.22 and 0.04 among SoC participants. Most studies are likely estimating suppression rates with these anti‐conservative assumptions, while the estimates we provided in our primary analysis may be much closer to the reality and could explain, at least in part, why transmission is still so high in, for example, Kwa Zulu Natal, South Africa, bordering Eswatini [30]. Calendar time was controlled for in all analyses, thus eliminating background time trends related to a general improvement in the quality of care as an explanation for the large observed increase in viral suppression among the EAAA group.

Lessons learned from this trial are as follows: EAAA can be successfully implemented in a public sector health system, supporting Eswatini and other countries' decisions to roll out this policy as the SoC. EAAA adoption did not lead to a deterioration of clinical performance of the public sector ART provision, as some had feared. Self‐reported adherence and disclosure levels remained high after the introduction of EAAA, and we also observed an improvement in the health care interactions, possibly due to training at participating facilities, which will be an important element for a successful rollout of immediate ART [31]. Thus, EAAA not only benefited those who are either newly diagnosed or enrolled in pre‐ART but also those who were eligible for ART prior to EAAA. EAAA may be a particularly useful policy for providing ART coverage to all people living with HIV while supporting the attainment of the UNAIDS 90‐90‐90 target. Even in this resource‐limited, public sector clinical setting, EAAA participants were initiated on ART 3.6 fold faster than SoC participants (*p* < 0.001). As countries implement ART scale‐up programmes, there is also a need for policies and interventions to sustain long‐term retention and viral suppression rates, such as increased access to viral monitoring for all participants, mobile phone text messages for appointment reminders and VL result delivery, and others [32, 33, 34, 35].

This trial had several limitations. The Eswatini national ART guidelines evolved during the trial from recommending ART initiation at CD4 ≤ 350 to CD4 ≤ 500 and finally to EAAA. These changes over time reduced the statistical power to demonstrate significant impact of EAAA compared with the SoC. However, because the effect sizes were of a greater magnitude than anticipated, we were nevertheless able to observe highly significant EAAA effects.

Another limitation of this trial resulted from a relatively high proportion of missing VL measurements. In the primary analysis, we treated all missing VLs as detectable. If VLs are not measured as per national guidelines, it is difficult to properly treat participants, correct treatment failure and monitor and address non‐adherence. Insufficient VL monitoring is equivalent to being unsuppressed from health systems, patient safety and disease transmission standpoints. Under the national guidelines, VLs are not measured until ART initiation and every six months thereafter. Thus, even if SoC and EAAA were compared contemporaneously, there would be more VLs in the EAAA group since EAAA participants are immediately initiated regardless of clinical status. However, the sensitivity analyses demonstrated that regardless of whether we included only those with at least one VL measurement after baseline, considered all EAAA participants without VLs as unsuppressed and SoC participants without VLs as suppressed, or considered all participants with missing VLs suppressed, results gave similarly attenuated but nevertheless significant improvements in suppression rates.

These real‐world limitations highlight the significant implementation challenges for the scale‐up of VL testing. The relatively poor performance in ensuring regular and timely VL testing is in line with results from public sector ART programmes in six other sub‐Saharan African countries [36]. Anecdotally reported reasons for the relatively poor VL testing performance included unavailability of daily sample transport, improper timing of sample pickups and a lack of centrifuge and plasma storage capabilities. Our trial was conducted under “real life” conditions. In contrast, at least two of the trials testing the effect of EAAA on HIV incidence have been carried out in a research‐grade clinical trial setting supported by dedicated trial staff. In these trials, VL testing occurred with adherence to recommended schedules that exceeded the level achieved in public‐sector provision in “real life” [3, 37]. The results from these studies could thus be considered closer to the efficacy end of the EAAA effect size spectrum, while our results could be considered closer to the effectiveness end of the spectrum.

## 5 Conclusions

Our findings provide strong causal evidence for the benefits of EAAA on retention and viral suppression, both for those who were already eligible for ART under the previous eligibility thresholds and for those who became newly eligible under EAAA. Thus, Eswatini and other countries with similar HIV epidemics and health systems should continue their rollout of EAAA. EAAA policies will likely not only improve access to ART but also the clinical performance of ART programmes with respect to patient outcomes.

## Competing Interests

All authors declare no competing interests.

## Authors’ Contributions

TB, VO, DS and FW (alphabetical order) designed the trial. AH, CL, SK, KM, EM, SM and MP (alphabetical order) implemented the trial. TB, AC, SK, DS and FW searched the literature and wrote the manuscript. AC, BC and DS (alphabetical order) conducted the statistical analysis and sample size calculations. TB, AC, WD, YF, AH, CL, GK, SK, KM, SM, VO, MP, RR, DS, FW and MZ (alphabetical order) contributed to the interpretation and presentation of the findings. All authors contributed to and approved the final version of the manuscript for submission.

## Data Safety Monitoring Board

Sten Vermund, USA (chair); Nhlanhla Nhlabatsi, Eswatini (co‐chair); Rhinos Chekenyere, Eswatini; Shaffiq Essajee, USA; Nomsa Mulima, Eswatini; and Candida Moshiro, Tanzania.

## Supporting information


**Figure S1.** Definition of non‐retention.
**Figure S2.** Definition of viral suppression.
**Figure S3.** Kaplan‐Meier curves for viral suppression. (a) all participants, (b) SoC ineligible and (c) SoC eligible.
**Figure S4.** Kaplan‐Meier curves for combined endpoint, retention and viral suppression. (a) all participants, (b) above SoC and (c) under SoC participants.
**Figure S5.** Forest plot of the intervention effect modification for retention, by covariates.
**Figure S6.** Forest plot of the intervention effect modification for retention, by covariates.
**Figure S7.** Forest plot of the intervention effect modification for viral suppression, by covariates.
**Figure S8.** Forest plot of the intervention effect modification for the combined endpoint, by covariates
**Talbe S1.** Effect of EAAA on retention among all participants, by pre‐specified subgroups.
**Table S2.** Effect of EAAA on viral suppression six months or more after ART initiation among all participants retained to ART initiation, by pre‐specified subgroups
**Table S3.** Effect of EAAA on retention and viral suppression among all participants, by pre‐specified subgroups
