# Supplementary material for: Early access to antiretroviral therapy versus standard of care among HIV‐positive participants in Eswatini in the public health sector: the MaxART stepped‐wedge randomized controlled trial
Source: J Int AIDS Soc. 2020 Sep 19;23(9):e25610. doi: 10.1002/jia2.25610 (PMC7507004; doi:10.1002/jia2.25610)
Supplement: Supplementary file 1 — Figure S1. Definition of non‐retention. Figure S2. Definition of viral suppression. Figure S3. Kaplan‐Meier curves for viral suppression. (a) all participants, (b) SoC ineligible and (c) SoC eligible. Figure S4. Kaplan‐Meier curves for combined endpoint, retention and viral suppression. (a) all participants, (b) above SoC and (c) under SoC participants. Figure S5. Forest plot of the intervention effect modification for retention, by covariates. Figure S6. Forest plot of the intervention effect modification for retention, by covariates. Figure S7. Forest plot of the intervention effect modification for viral suppression, by covariates. Figure S8. Forest plot of the intervention effect modification for the combined endpoint, by covariates Talbe S1. Effect of EAAA on retention among all participants, by pre‐specified subgroups. Table S2. Effect of EAAA on viral suppression six months or more after ART initiation among all participants retained to ART initiation, by pre‐specified subgroups Table S3. Effect of EAAA on retention and viral suppression among all participants, by pre‐specified subgroups [file JIA2-23-e25610-s001.docx]

## APPENDIX


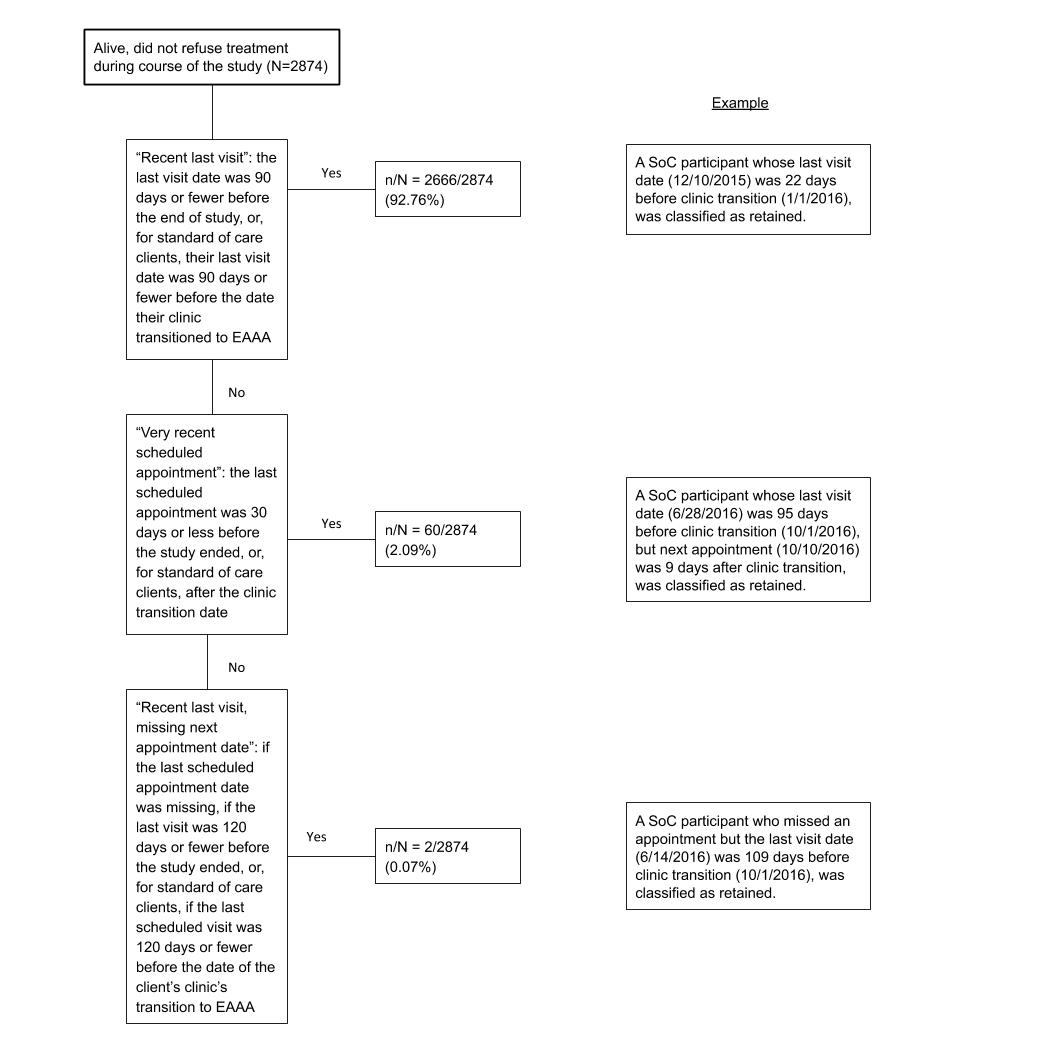


In addition, there were 146/2874 (5.08%) participants who transferred out of MaxART clinics, but were alive and did not stop treatment. They were considered retained, up to the point they transferred out and were then censored.

Supplementary Figure 1. Definition of non-retention


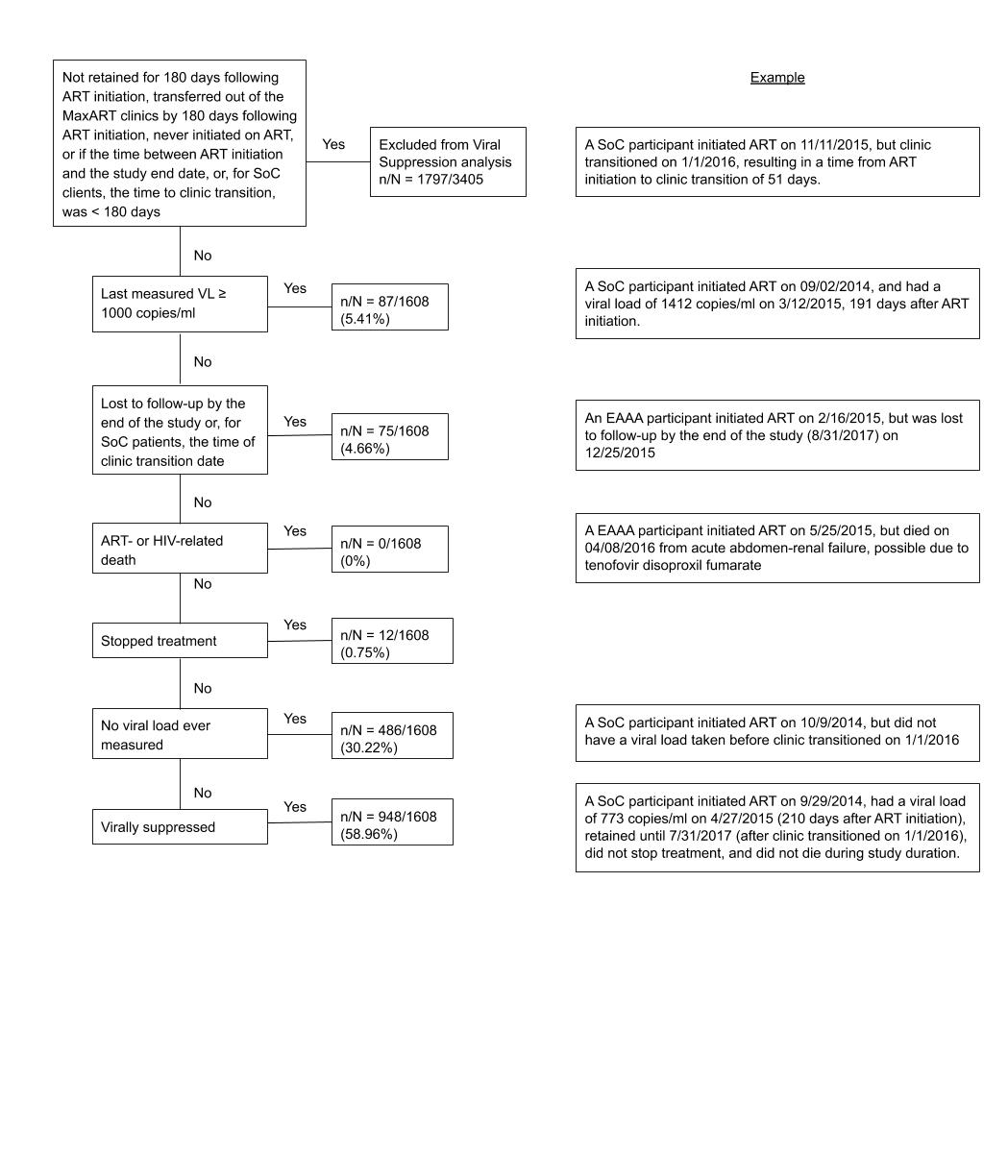
Supplementary Figure 2. Definition of viral suppression

## 3

**A) All participants (n/N=948/1608)**

**B) Above SoC (n/N=300/479)**

**C) Under SoC (n/N=585/1015)**


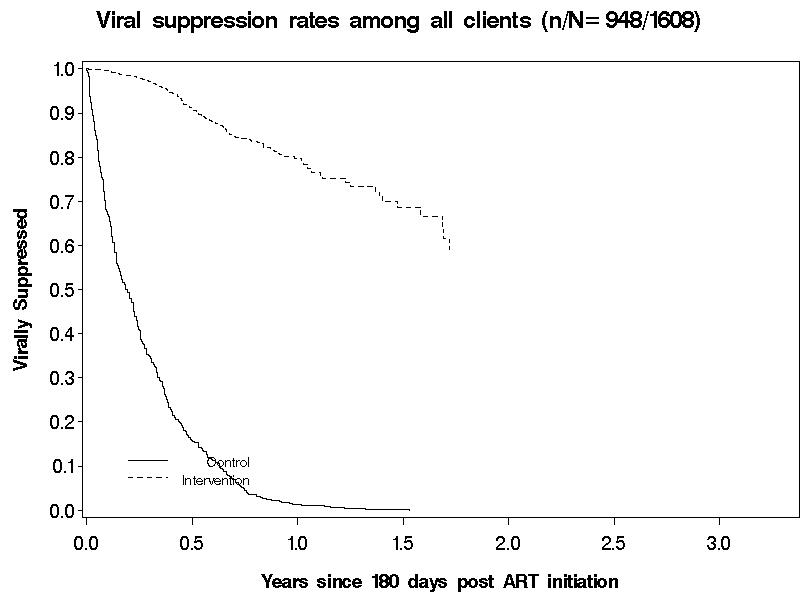

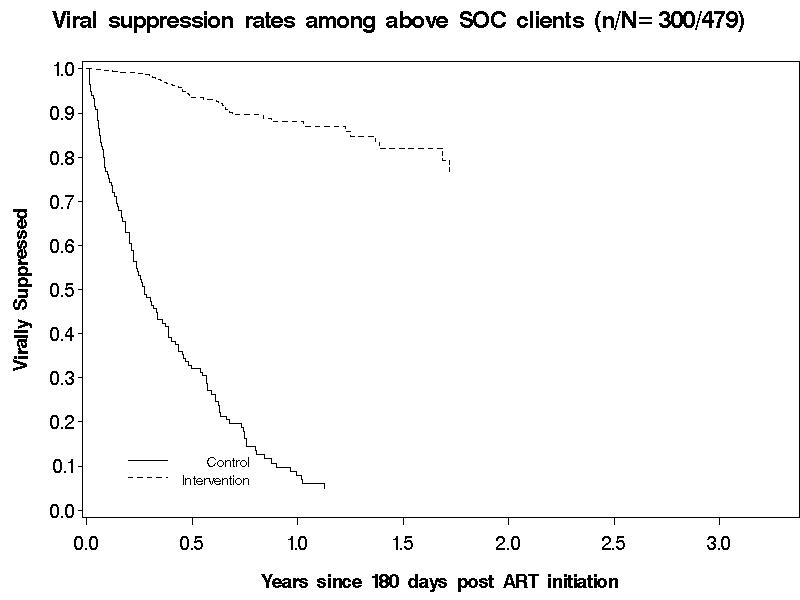

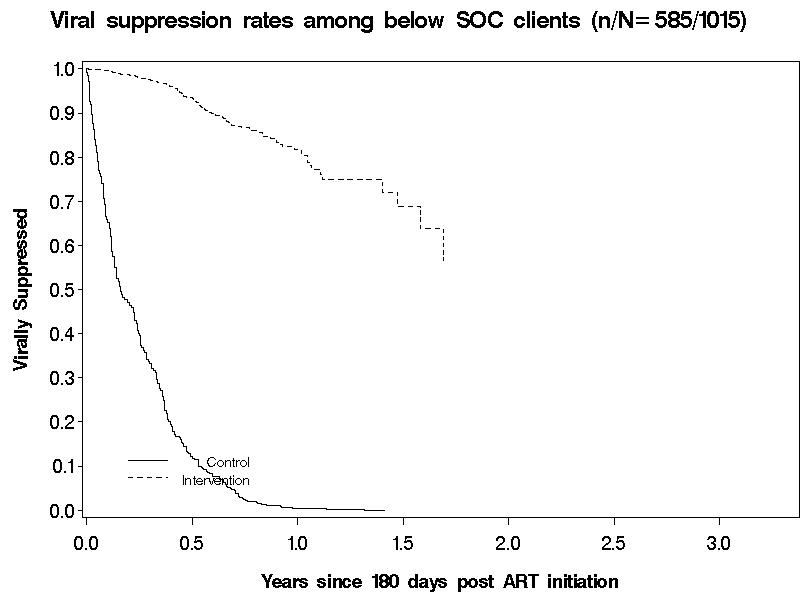


**Supplementary Figure 3: Kaplan-Meier curves for viral suppression. a) all participants, b) SoC ineligible and c) SoC eligible.** Graphs reflect the cumulative incidence averaged over covariates. Full multivariable model adjusted for steptime, age (18-<20 yr old, 20-<30 yr old,30-<40 yr old, 40-<50 yr old,50-<60 yr old, 60+ yr old), sex, marital status (Married, Divorced/Widowed, Single), education (Illiterate/Primary, Secondary, High School, Tertiary), CD4 at 180 days post ART initiation (<350, 350-500, >500), WHO stage at 180 days post ART initiation (stage 1, stage 2, stage 3 or 4), BMI (<18.5, 18.5-<25,25-<30, ≥30), screened for TB symptoms (yes, no), viral load (<5‚000, 5‚000-30‚000, >30‚000), access to HIV Treatment supporter (yes, no), level of clinic (Hospital, Clinic with maternity, Clinic without maternity), time from HIV tested positive to enrollment (≤1 yr,1- ≤3 yr,>3 yr), clinic volume (Low: < median, High: ≥ median), study enrollment date (2 knots SRCS). If CD4/WHO at 180 days post ART initiation is not available, used CD4/WHO at ART initiation if available, or CD4/WHO at study enrollment if available. For all participants and SoC eligible subgroup, WHO stage at 180 days post ART initiation had too few missing values to be a category and the missing was assigned to a common category.

**A) All participants (n/N=2166/3405)**

**B) SoC ineligible (n/N=840/1225)**

**C) SoC eligible (n/N=1044/1754)**


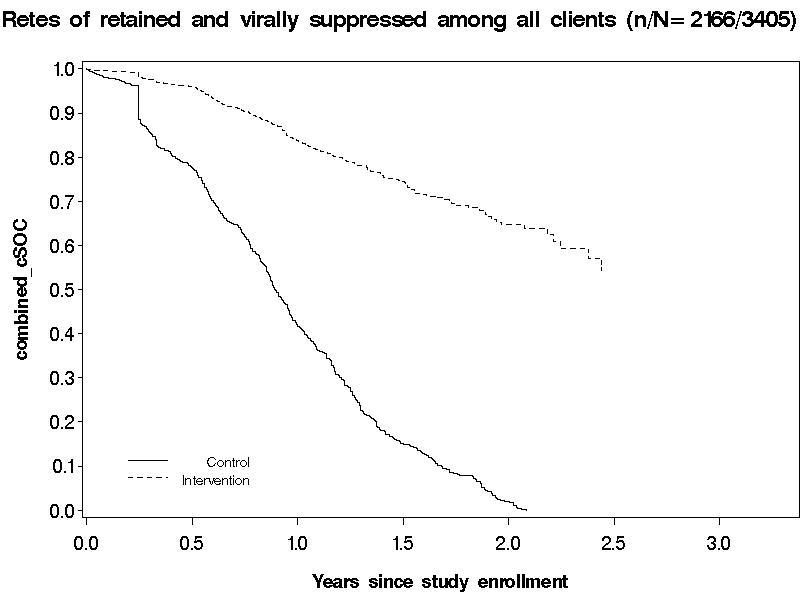

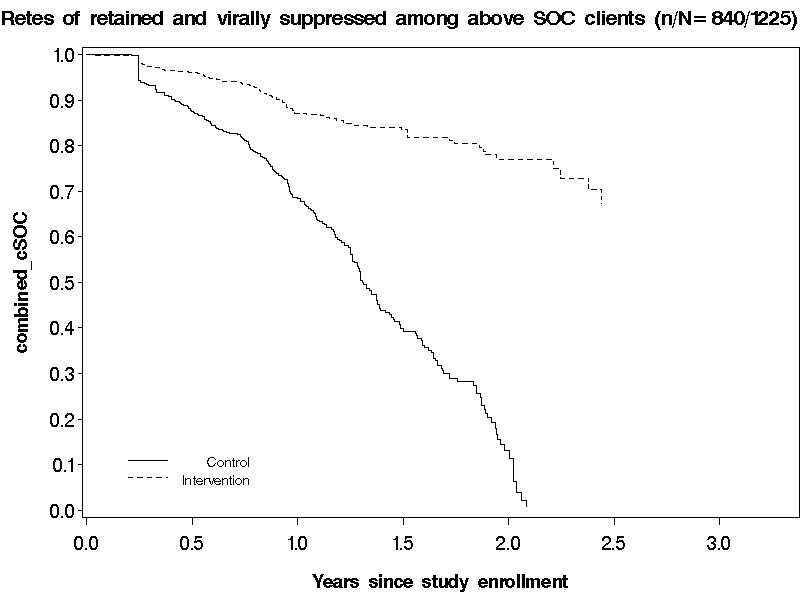

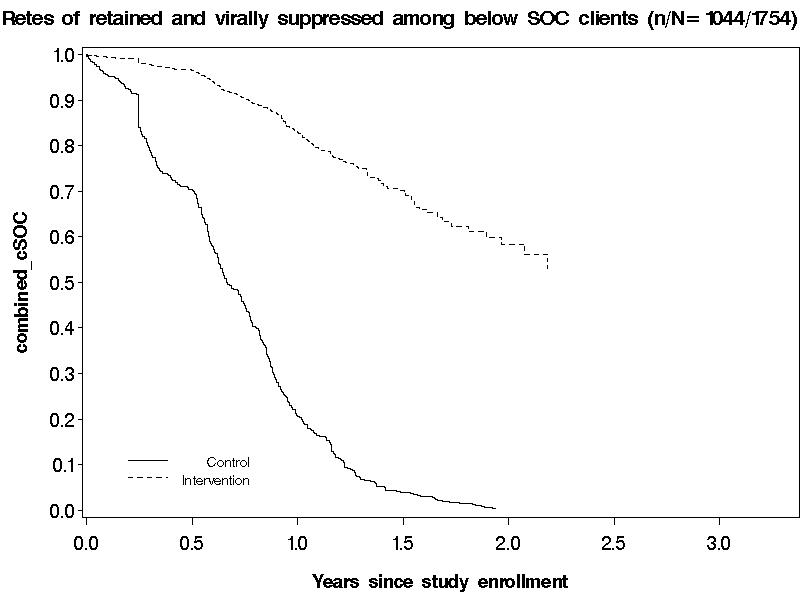


**Supplementary Figure 4: Kaplan-Meier curves for combined endpoint, retention and viral suppression. a) all participants, b) above SoC and c) under SoC participants.** Graphs reflect the cumulative incidence averaged over covariates. Full multivariable model adjusted for steptime, age (18-<20 yr old, 20-<30 yr old,30-<40 yr old, 40-<50 yr old,50-<60 yr old, 60+ yr old), sex, marital status (Married, Divorced/Widowed, Single), education (Illiterate/Primary, Secondary, High School, Tertiary), CD4 (<350, 350-500, >500), WHO stage (stage 1, stage 2, stage 3 or 4), BMI (<18.5, 18.5-<25,25-<30, ≥30), screened for TB symptoms (yes, no), viral load (<5‚000, 5‚000-30‚000, >30‚000), access to HIV Treatment supporter (yes, no), level of clinic (Hospital, Clinic with maternity, Clinic without maternity), time from HIV tested positive to enrollment (≤1 yr,1- ≤3 yr,>3 yr), clinic volume (Low: < median, High: ≥ median), study enrollment date (continuous). All participants graph included study enrolment date (2 knots SRCS), SOC eligible graph included study enrollment date (3 knots SRCS), and SoC ineligible included study enrolment date


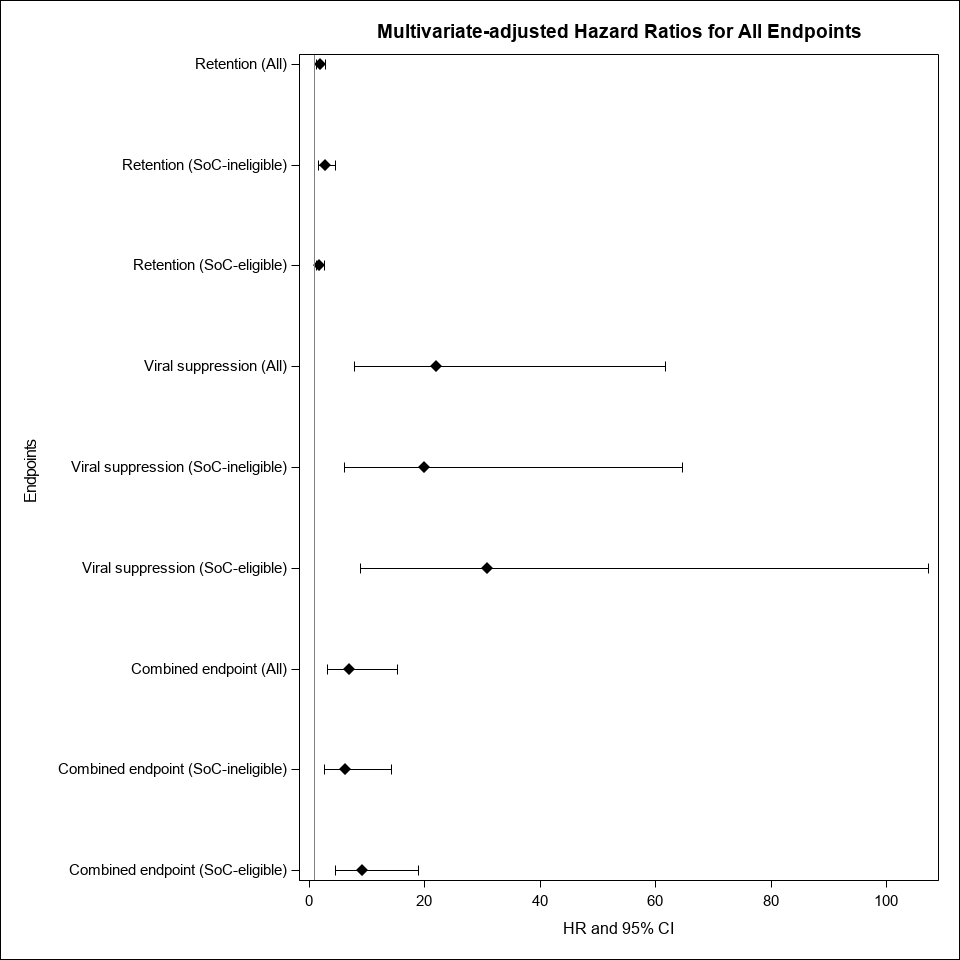


Supplementary Figure 5. Forest plot of interaction effects for three endpoints: all enrolled subjects, SoC-ineligible and SoC-eligible, multivariable-adjusted


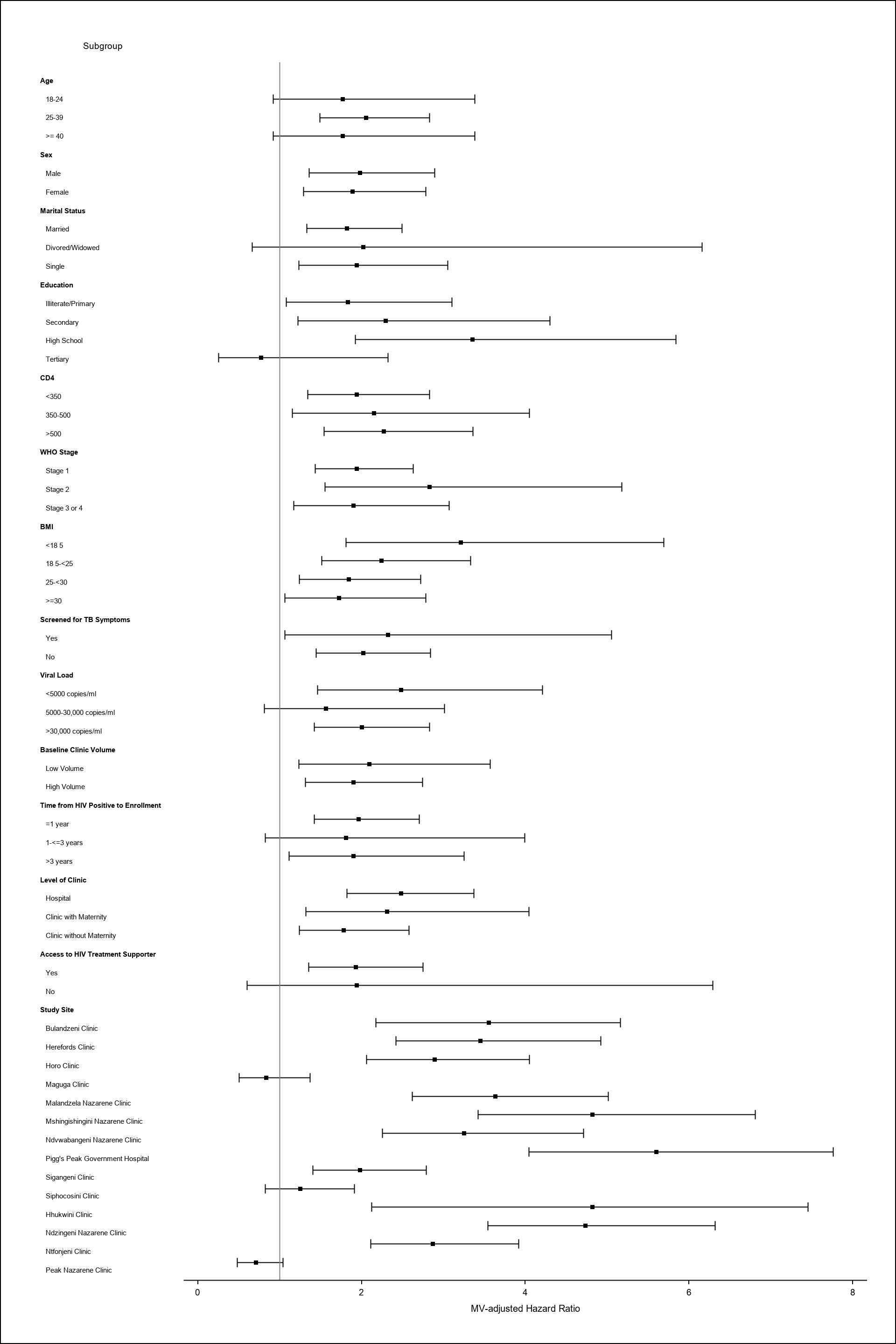


Supplementary Figure 6. Forest plot of the intervention effect modification for retention, by covariates


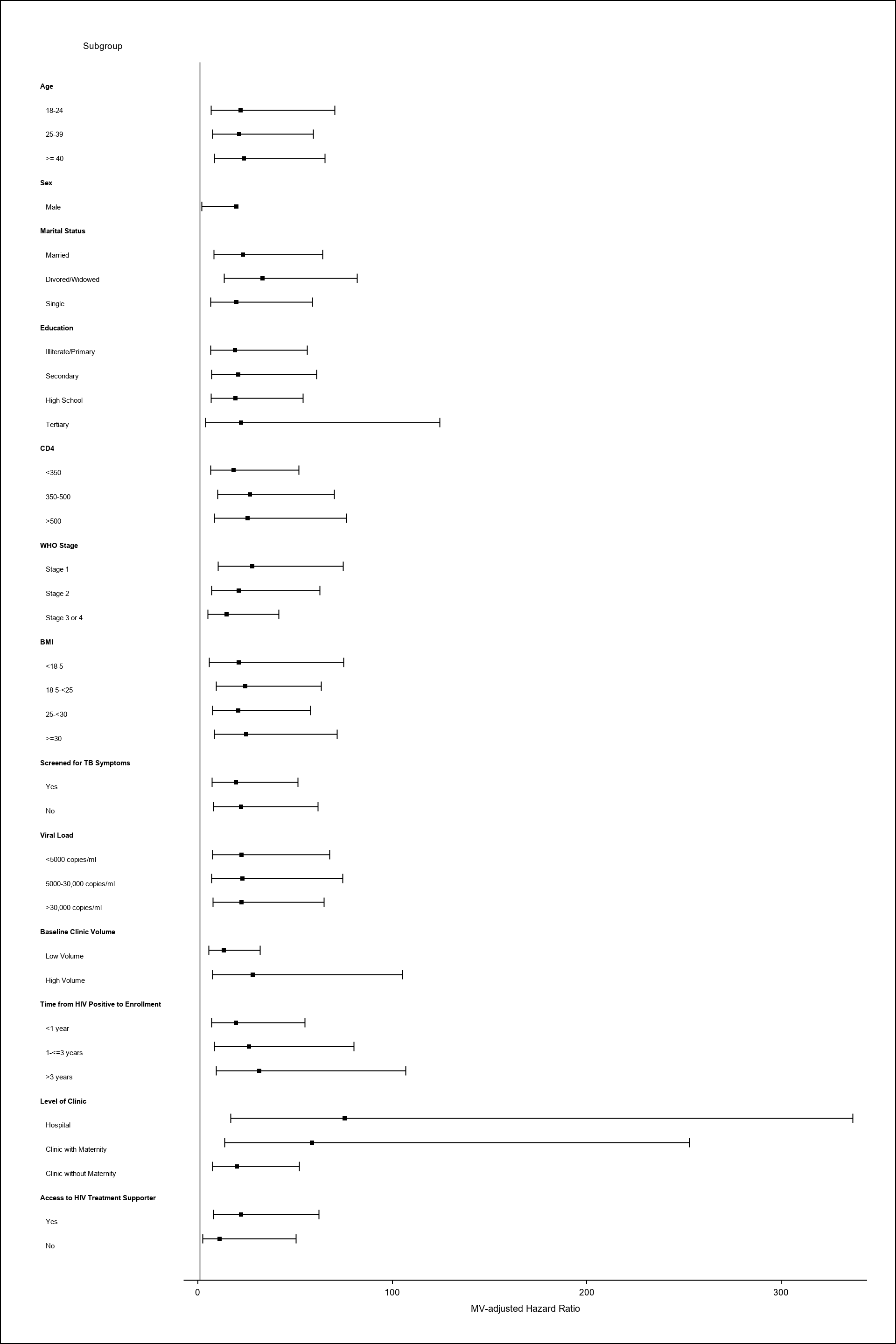


Supplementary Figure 7. Forest plot of the intervention effect modification for viral suppression, by covariates


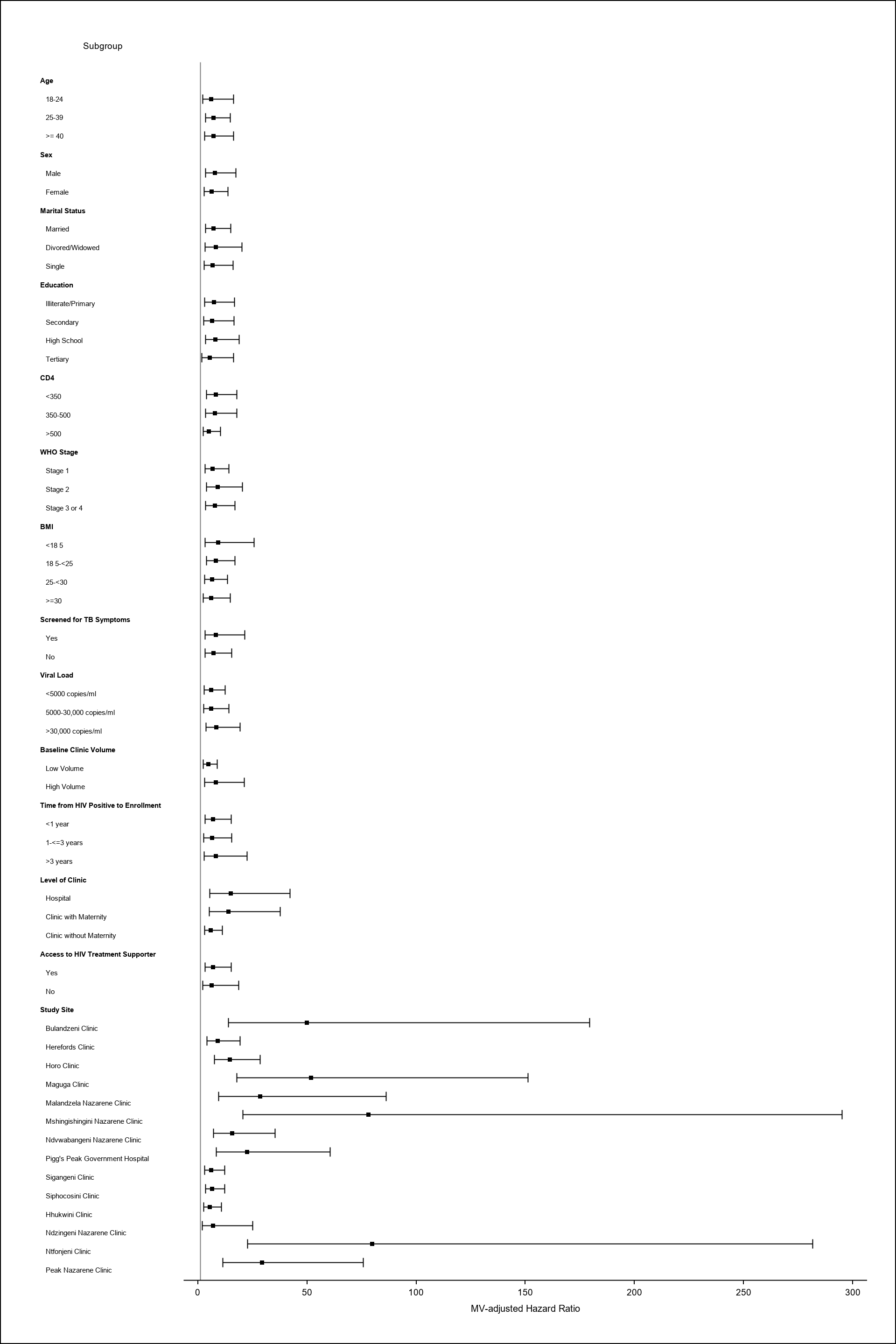


Supplementary Figure 8. Forest plot of the intervention effect modification for the combined endpoint, by covariates

**Supplementary Table 1. Effect of EAAA on retention among all participants, by pre-specified subgroups**

| **Subgroup** | **Standard of Care** | | | **EAAA** | | |  |  |
| --- | --- | --- | --- | --- | --- | --- | --- | --- |
|  | **n/N** | **Follow-up time-person year** | **Non-retention rate-person year** | **n/N** | **Follow-up time-person year** | **Non-retention rate-person year** | **Multivariable HR**^1,2,3^ **(95%CI)** | **p-value, test for heterogeneity**^2,4^ |
| **Age** | | | |  |  |  |  | 0.88 |
| 18-24 | 63/293 | 216.8 | 0.29 | 48/211 | 188.9 | 0.25 | 1.77 (0.92-3.38) |  |
| 25-39 | 181/1148 | 910.3 | 0.20 | 122/795 | 798.4 | 0.15 | 2.05 (1.49-2.83) |  |
| ≥40 | 72/593 | 538.6 | 0.13 | 45/365 | 377.3 | 0.12 | 1.77 (0.92-3.38) |  |
| **Sex** |  |  |  |  |  |  |  | 0.73 |
| Male | 116/695 | 492.0 | 0.24 | 99/603 | 581.4 | 0.17 | 1.98 (1.36-2.89) |  |
| Female | 200/1339 | 1173.6 | 0.17 | 116/768 | 783.2 | 0.15 | 1.89 (1.29-2.78) |  |
| **Marital Status** | | | |  |  |  |  | 0.99 |
| Married | 123/1045 | 899.3 | 0.14 | 80/634 | 668.7 | 0.12 | 1.82 (1.33-2.49) |  |
| Divorced/ Widowed | 22/127 | 112.2 | 0.20 | 12/78 | 72.2 | 0.17 | 2.02 (0.66-6.16) |  |
| Single | 163/825 | 631.5 | 0.26 | 117/614 | 584.9 | 0.20 | 1.94 (1.23-3.05) |  |
| **Education** | | | |  |  |  |  | 0.11 |
| Illiterate/ Primary | 93/589 | 467.2 | 0.20 | 66/384 | 409.1 | 0.16 | 1.83 (1.08-3.10) |  |
| Secondary | 72/438 | 352.7 | 0.20 | 52/362 | 365.7 | 0.14 | 2.29 (1.22-4.30) |  |
| High School | 63/401 | 342.8 | 0.18 | 22/218 | 211.2 | 0.10 | 3.35 (1.92-5.84) |  |
| Tertiary | 5/48 | 34.2 | 0.15 | 9/37 | 30.1 | 0.30 | 0.77 (0.25-2.32) |  |
| **CD4^5^** | | | |  |  |  |  | 0.0006 |
| <350 | 121/804 | 596.9 | 0.20 | 94/632 | 662.8 | 0.14 | 1.94 (1.34-2.83) |  |
| 350-500 | 63/441 | 377.7 | 0.17 | 31/224 | 260.0 | 0.12 | 2.15 (1.15-4.05) |  |
| >500 | 101/591 | 536.1 | 0.19 | 39/266 | 309.0 | 0.13 | 2.27 (1.54-3.36) |  |
| **WHO stage^5^** | | | |  |  |  |  | 0.10 |
| stage 1 | 150/1074 | 900.6 | 0.17 | 116/800 | 771.3 | 0.15 | 1.94 (1.43-2.63) |  |
| stage 2 | 52/357 | 239.0 | 0.22 | 36/320 | 340.2 | 0.11 | 2.83 (1.55-5.18) |  |
| stage 3 or 4 | 54/264 | 181.2 | 0.30 | 31/146 | 168.6 | 0.18 | 1.90 (1.17-3.07) |  |
| **BMI ^5^** | | | |  |  |  |  | 0.03 |
| <18.5 | 19/82 | 52.3 | 0.36 | 11/81 | 66.9 | 0.16 | 3.21 (1.81-5.69) |  |
| 18.5-<25 | 161/971 | 765.4 | 0.21 | 109/762 | 748.3 | 0.15 | 2.24 (1.51-3.33) |  |
| 25-<30 | 74/529 | 439.9 | 0.17 | 44/281 | 288.9 | 0.15 | 1.84 (1.24-2.72) |  |
| ≥30 | 44/387 | 367.1 | 0.12 | 27/212 | 242.6 | 0.11 | 1.72 (1.06-2.78) |  |
| **Screened for TB symptoms^5^** | | | |  |  |  |  | 0.57 |
| Yes | 41/190 | 128.6 | 0.32 | 16/100 | 86.2 | 0.19 | 2.32 (1.06-5.05) |  |
| No | 250/1762 | 1486.8 | 0.17 | 171/1205 | 1236.6 | 0.14 | 2.02 (1.44-2.84) |  |
| **Viral load ^5^** | | | |  |  |  |  | 0.78 |
| <5000 Copies/ml | 74/435 | 370.0 | 0.20 | 34/228 | 238.6 | 0.14 | 2.48 (1.46-4.21) |  |
| 5000-30,000 copies/ml | 57/430 | 357.4 | 0.16 | 43/258 | 274.8 | 0.16 | 1.56 (0.81-3.01) |  |
| >30,000 copies/ml | 158/1019 | 838.6 | 0.19 | 103/701 | 692.6 | 0.15 | 2.00 (1.42-2.83) |  |
| **Baseline Clinic Volume^6^** | | |  |  |  |  |  | 0.68 |
| Low Volume | 129/915 | 876.2 | 0.15 | 35/385 | 282.0 | 0.12 | 2.09 (1.23-3.57) |  |
| High volume | 187/1119 | 789.5 | 0.24 | 180/986 | 1082.6 | 0.17 | 1.90 (1.31-2.74) |  |
| **Time from HIV positive to enrollment** | | | |  |  |  |  | 0.86 |
| ≤1 yr, %(n) | 189/1189 | 847.8 | 0.22 | 171/1076 | 973.3 | 0.18 | 1.96 (1.42-2.70) |  |
| 1- ≤3 yr, %(n) | 64/421 | 428.9 | 0.15 | 18/134 | 190.1 | 0.09 | 1.81 (0.82-3.99) |  |
| >3 yr, %(n) | 61/413 | 382.9 | 0.16 | 22/144 | 191.7 | 0.11 | 1.90 (1.11-3.25) |  |
| **Level of clinic** | |  |  |  |  |  |  | 0.14 |
| Hospital, %(n) | 96/449 | 283.7 | 0.34 | 64/349 | 285.2 | 0.22 | 2.48 (1.82-3.37) |  |
| Clinic with maternity, %(n) | 36/317 | 269.4 | 0.13 | 21/181 | 177.3 | 0.12 | 2.31 (1.32-4.04) |  |
| Clinic without maternity, %(n) | 184/1268 | 1112.6 | 0.17 | 130/841 | 902.1 | 0.14 | 1.78 (1.24-2.58) |  |
| **Access to HIV Treatment supporter** | |  |  |  |  |  |  | 0.99 |
| Yes | 308/1980 | 1625.9 | 0.19 | 209/1339 | 1329.6 | 0.16 | 1.93 (1.35-2.75) |  |
| No | 8/54 | 39.8 | 0.20 | 6/32 | 35.0 | 0.17 | 1.94 (0.60-6.29) |  |
| **Site** |  |  |  |  |  |  |  | 0.53 |
| Bulandzeni Clinic | 11/49 | 19.4 | 0.57 | 26/123 | 163.1 | 0.16 | 3.55 (2.17-5.16) |  |
| Herefords Clinic | 31/168 | 154.0 | 0.20 | 8/73 | 61.2 | 0.13 | 3.45 (2.42-4.92) |  |
| Horo Clinic | 57/313 | 369.8 | 0.15 | 2/44 | 20.7 | 0.10 | 2.89 (2.06-4.05) |  |
| Maguga Clinic | 9/110 | 58.5 | 0.15 | 18/116 | 137.3 | 0.13 | 0.83 (0.50-1.37) |  |
| Malandzela Nazarene Clinic | 4/50 | 29.3 | 0.14 | 12/57 | 56.4 | 0.21 | 3.63 (2.62-5.01) |  |
| Mshingishingini Nazarene Clinic | 6/81 | 17.1 | 0.35 | 19/119 | 160.0 | 0.12 | 4.82 (3.42-6.81) |  |
| Ndvwabangeni Nazarene Clinic | 27/207 | 210.9 | 0.13 | 3/65 | 40.1 | 0.07 | 3.25 (2.25-4.71) |  |
| Pigg's Peak Government Hospital | 96/449 | 283.7 | 0.34 | 64/349 | 285.2 | 0.22 | 5.60 (4.04-7.76) |  |
| Sigangeni Clinic | 12/159 | 174.9 | 0.07 | 7/52 | 28.0 | 0.25 | 1.98 (1.40-2.79) |  |
| Siphocosini Clinic | 40/203 | 189.6 | 0.21 | 5/55 | 29.0 | 0.17 | 1.25 (0.82-1.91) |  |
| Hhukwini Clinic | 15/97 | 99.6 | 0.15 | 2/23 | 13.6 | 0.15 | 4.82 (2.12-7.45) |  |
| Ndzingeni Nazarene Clinic | 1/40 | 16.5 | 0.06 | 9/82 | 87.6 | 0.10 | 4.73 (3.54-6.32) |  |
| Ntfonjeni Clinic | 4/67 | 11.7 | 0.34 | 39/178 | 260.0 | 0.15 | 2.87 (2.11-3.92) |  |
| Peak Nazarene Clinic | 3/41 | 30.7 | 0.10 | 1/35 | 22.6 | 0.04 | 0.71 (0.48-1.04) |  |

^1^ Adjusted for steptime, age (18-24, 25-39, ≥40 years), sex, marital status (Married, Divorced/Widowed, Single), education (Illiterate/Primary, Secondary, High School, Tertiary), CD4 counts (<350, 350-500, >500), WHO stAge (stage 1, stage 2, stage 3 or 4), BMI (<18.5, 18.5-<25,25-<30, ≥30), Screened for TB symptoms (yes, no), viral load (<5‚000, 5‚000-30‚000, >30‚000), treatment support (yes, no), Level of clinic (Hospital, Clinic with maternity, Clinic without maternity), time from HIV tested positive to enrollment (≤1 yr,1- ≤3 yr,>3 yr), clinic volume (Low: < median, High: ≥ median), study enrollment date (continuous). Missing data was treated as a separate group for each of the covariates in the models, except for marital status and time from HIV tested positive to enrollment, which have too few missing to be a category and the missing was assigned to common category.

^2^ Confidence intervals not adjusted for multiplicity

^3^ Reference group = Standard of Care.

^4^ Robust Wald test accounting for clustering for interaction. Median scores were used for Age, CD4, BMI , viral load .

^5^ Within 90 days of enrollment and before ART initiation.

- ^6^ First quarter into study

**Supplementary Table 2. Effect of EAAA on viral suppression 6 months or more after ART initiation among all participants retained to ART initiation, by pre-specified subgroups**

| **Subgroup** | **Standard of Care** | | | **EAAA** | | |  |  |
| --- | --- | --- | --- | --- | --- | --- | --- | --- |
|  | **n/N** | **Follow-up time-person year** | **Treatment failure rate-person year** | **n/N** | **Follow-up time-person year** | **Treatment failure rate-person year** | **Multivariable HR**^1,2,3^ **(95%CI)** | **p-value, test for heterogeneity**^2,4^ |
| **Age** | | | |  |  |  |  | 0.61 |
| 18-24 | 41/75 | 33.4 | 1.23 | 60/132 | 84.2 | 0.71 | 21.73 (6.72-70.28) |  |
| 25-39 | 209/415 | 227.3 | 0.92 | 177/516 | 408.8 | 0.43 | 21.17 (7.55-59.33) |  |
| ≥40 | 102/223 | 126.2 | 0.81 | 71/247 | 198.1 | 0.36 | 23.55 (8.49-65.29) |  |
| **Sex** |  |  |  |  |  |  |  | 0.17 |
| Male | 122/250 | 136.4 | 0.89 | 145/387 | 286.5 | 0.51 | 19.79 (6.65-58.91) |  |
| Female | 230/463 | 250.5 | 0.92 | 163/508 | 404.6 | 0.40 | 23.37 (8.59-63.57) |  |
| **Marital Status** | | | |  |  |  |  | 0.20 |
| Married | 192/394 | 220.4 | 0.87 | 134/436 | 345.1 | 0.39 | 23.01 (8.25-64.15) |  |
| Divorced/ Widowed | 30/54 | 29.4 | 1.02 | 16/49 | 37.3 | 0.43 | 33.20 (13.47-81.86) |  |
| Single | 122/255 | 133.9 | 0.91 | 146/383 | 289.0 | 0.51 | 19.63 (6.56-58.74) |  |
| **Education** | | | |  |  |  |  | 0.84 |
| Illiterate/ Primary | 93/195 | 104.7 | 0.89 | 83/256 | 214.3 | 0.39 | 19.04 (6.46-56.14) |  |
| Secondary | 80/157 | 83.9 | 0.95 | 96/251 | 184.1 | 0.52 | 20.65 (6.99-61.04) |  |
| High School | 70/153 | 96.4 | 0.73 | 52/143 | 106.0 | 0.49 | 19.21 (6.84-54.00) |  |
| Tertiary | 10/18 | 8.3 | 1.21 | 11/21 | 13.8 | 0.80 | 22.01 (3.90-124.23) |  |
| **CD4^5^** | | | |  |  |  |  | 0.12 |
| <350 | 183/368 | 214.1 | 0.85 | 154/395 | 288.6 | 0.53 | 18.21 (6.39-51.88) |  |
| 350-500 | 85/166 | 88.1 | 0.97 | 49/174 | 142.2 | 0.34 | 26.72 (10.19-70.06) |  |
| >500 | 80/173 | 81.1 | 0.99 | 78/269 | 238.6 | 0.33 | 25.39 (8.45-76.31) |  |
| **WHO stage^6^** | | | |  |  |  |  | 0.004 |
| stage 1 | 184/379 | 200.9 | 0.92 | 160/467 | 333.0 | 0.48 | 27.75 (10.33-74.55) |  |
| stage 2 | 96/188 | 100.3 | 0.96 | 91/287 | 246.3 | 0.37 | 20.89 (6.96-62.71) |  |
| stage 3 or 4 | 72/146 | 85.7 | 0.84 | 57/141 | 111.8 | 0.51 | 14.63 (5.16-41.47) |  |
| **BMI ^7^** | | | |  |  |  |  | 0.04 |
| <18.5 | 18/32 | 13.4 | 1.35 | 26/50 | 30.6 | 0.85 | 20.87 (5.81-75.00) |  |
| 18.5-<25 | 178/355 | 192.8 | 0.92 | 161/500 | 387.8 | 0.42 | 24.23 (9.27-63.35) |  |
| 25-<30 | 88/190 | 106.5 | 0.83 | 69/188 | 140.3 | 0.49 | 20.63 (7.35-57.86) |  |
| ≥30 | 66/128 | 67.9 | 0.97 | 48/152 | 129.3 | 0.37 | 24.61 (8.48-71.42) |  |
| **Screened for TB symptoms^7^** | | | |  |  |  |  | 0.35 |
| Yes | 39/76 | 37.1 | 1.05 | 19/55 | 39.2 | 0.49 | 19.36 (7.31-51.25) |  |
| No | 303/623 | 343.5 | 0.88 | 279/819 | 641.2 | 0.44 | 22.03 (7.85-61.79) |  |
| **Viral load ^7^** | | | |  |  |  |  | 0.74 |
| <5000 Copies/ml | 39/93 | 47.2 | 0.83 | 39/140 | 124.1 | 0.31 | 22.39 (7.42-67.58) |  |
| 5000-30,000 copies/ml | 73/141 | 73.1 | 1.00 | 55/171 | 148.9 | 0.37 | 22.73 (6.94-74.51) |  |
| >30,000 copies/ml | 214/429 | 239.6 | 0.89 | 169/478 | 341.3 | 0.50 | 22.34 (7.71-64.71) |  |
| **Baseline Clinic Volume^8^** | | |  |  |  |  |  | 0.39 |
| Low Volume | 171/377 | 218.2 | 0.78 | 112/231 | 116.1 | 0.96 | 13.20 (5.45-31.97) |  |
| High volume | 181/336 | 168.8 | 1.07 | 196/664 | 575.0 | 0.34 | 28.05 (7.48-105.19) |  |
| **Time from HIV positive to enrollment** | | | |  |  |  |  | 0.12 |
| ≤1 yr, %(n) | 207/421 | 231.0 | 0.90 | 249/671 | 460.2 | 0.54 | 19.40 (6.86-54.87) |  |
| 1- ≤3 yr, %(n) | 79/152 | 80.4 | 0.98 | 30/104 | 115.9 | 0.26 | 26.09 (8.49-80.19) |  |
| >3 yr, %(n) | 65/139 | 75.4 | 0.86 | 24/112 | 112.8 | 0.21 | 31.46 (9.27-106.81) |  |
| **Level of clinic** | |  |  |  |  |  |  | 0.0001 |
| Hospital, %(n) | 80/122 | 53.5 | 1.49 | 82/211 | 128.9 | 0.64 | 75.40 (16.88-336.75) |  |
| Clinic with maternity, %(n) | 60/112 | 52.5 | 1.14 | 31/121 | 85.0 | 0.36 | 58.59 (13.58-252.74) |  |
| Clinic without maternity, %(n) | 212/479 | 280.9 | 0.75 | 195/563 | 477.2 | 0.41 | 19.82 (7.55-52.07) |  |
| **Access to HIV Treatment supporter** | |  |  |  |  |  |  | 0.27 |
| Yes | 347/697 | 375.6 | 0.92 | 302/874 | 671.2 | 0.45 | 22.12 (7.87-62.21) |  |
| No | 5/16 | 11.4 | 0.44 | 6/21 | 19.9 | 0.30 | 10.94 (2.37-50.48) |  |

^1^ Adjusted for steptime, Age (18-24, 25-39, ≥40 years), sex, marital status (Married, Divorced/Widowed, Single), education (Illiterate/Primary, Secondary, High School, Tertiary), CD4 counts (<350, 350-500, >500), WHO stAge (stage 1, stage 2, stage 3 or 4), BMI (<18.5, 18.5-<25,25-<30, ≥30), Screened for TB symptoms (yes, no), viral load (<5‚000, 5‚000-30‚000, >30‚000), treatment support (yes, no), Level of clinic (Hospital, Clinic with maternity, Clinic without maternity), time from HIV tested positive to enrollment (≤1 yr,1- ≤3 yr,>3 yr), clinic volume (Low: < median, High: ≥ median), study enrollment date (continuous). Missing data was treated as a separate group for each of the covariates in the models, except for marital status and time from HIV tested positive to enrollment, which have too few missing to be a category and the missing was assigned to common category.

^2^ Confidence intervals not adjusted for multiplicity

^3^ Reference group = Standard of Care.

^4^ Robust Wald test accounting for clustering for interaction. Median scores were used for Age, CD4, BMI, and viral load.

^5^ Within 90 days of enrollment and before ART initiation.

- ^6^ First quarter into study

**Supplementary Table 3. Effect of EAAA on retention and viral suppression among all participants, by pre-specified subgroups**

| **Subgroup** | **Standard of Care** | | | **EAAA** | | |  |  |
| --- | --- | --- | --- | --- | --- | --- | --- | --- |
|  | **n/N** | **Follow-up time-person year** | **Non-retention/Treatment failure rate-person year** | **n/N** | **Follow-up time-person year** | **Non-retention/Treatment failure rate-person year** | **Multivariable HR**^1,2,3^ **(95%CI)** | **p-value, test for heterogeneity** ^2,4^ |
| **Age** | | | |  |  |  |  | 0.48 |
| 18-24 | 115/293 | 215.6 | 0.53 | 105/211 | 185.1 | 0.57 | 5.96 (2.19-16.24) |  |
| 25-39 | 417/1148 | 900.5 | 0.46 | 299/795 | 782.3 | 0.38 | 7.04 (3.34-14.85) |  |
| ≥40 | 191/593 | 533.7 | 0.36 | 112/365 | 364.8 | 0.31 | 7.10 (3.08-16.35) |  |
| **Sex** |  |  |  |  |  |  |  | 0.005 |
| Male | 268/695 | 488.7 | 0.55 | 234/603 | 564.9 | 0.41 | 7.76 (3.46-17.42) |  |
| Female | 455/1339 | 1161.1 | 0.39 | 282/768 | 767.3 | 0.37 | 6.29 (2.88-13.72) |  |
| **Marital Status** | | | |  |  |  |  | 0.45 |
| Married | 343/1045 | 890.8 | 0.39 | 214/634 | 653.3 | 0.33 | 7.06 (3.33-14.98) |  |
| Divorced/ Widowed | 49/127 | 108.9 | 0.45 | 27/78 | 70.1 | 0.39 | 8.14 (3.28-20.20) |  |
| Single | 313/825 | 627.5 | 0.50 | 255/614 | 572.5 | 0.45 | 6.59 (2.72-15.94) |  |
| **Education** | | | |  |  |  |  | 0.32 |
| Illiterate/ Primary | 214/589 | 463.8 | 0.46 | 141/384 | 399.6 | 0.35 | 7.19 (3.09-16.77) |  |
| Secondary | 154/438 | 351.0 | 0.44 | 145/362 | 353.2 | 0.41 | 6.41 (2.50-16.42) |  |
| High School | 137/401 | 339.6 | 0.40 | 77/218 | 209.0 | 0.37 | 7.95 (3.37-18.75) |  |
| Tertiary | 19/48 | 33.7 | 0.56 | 21/37 | 29.1 | 0.72 | 5.40 (1.79-16.32) |  |
| **CD4^5^** | | | |  |  |  |  | 0.26 |
| <350 | 347/804 | 585.2 | 0.59 | 242/632 | 639.9 | 0.38 | 8.21 (3.81-17.67) |  |
| 350-500 | 156/441 | 374.3 | 0.42 | 73/224 | 257.3 | 0.28 | 7.80 (3.43-17.75) |  |
| >500 | 168/591 | 535.3 | 0.31 | 100/266 | 304.7 | 0.33 | 4.86 (2.32-10.19) |  |
| **WHO stage^5^** | | | |  |  |  |  | 0.006 |
| stage 1 | 359/1074 | 893.4 | 0.40 | 285/800 | 761.2 | 0.37 | 6.72 (3.19-14.19) |  |
| stage 2 | 134/357 | 234.3 | 0.57 | 111/320 | 330.9 | 0.34 | 8.88 (3.87-20.34) |  |
| stage 3 or 4 | 124/264 | 178.0 | 0.70 | 68/146 | 157.8 | 0.43 | 7.71 (3.50-16.96) |  |
| **BMI ^5^** | | | |  |  |  |  | 0.03 |
| <18.5 | 39/82 | 51.1 | 0.76 | 34/81 | 63.7 | 0.53 | 9.19 (3.30-25.59) |  |
| 18.5-<25 | 363/971 | 758.6 | 0.48 | 264/762 | 735.1 | 0.36 | 8.04 (3.84-16.85) |  |
| 25-<30 | 180/529 | 437.2 | 0.41 | 114/281 | 280.0 | 0.41 | 6.42 (3.09-13.38) |  |
| ≥30 | 119/387 | 362.1 | 0.33 | 74/212 | 235.5 | 0.31 | 5.95 (2.39-14.80) |  |
| **Screened for TB symptoms^5**^** | | | |  |  |  |  | 0.36 |
| Yes | 86/190 | 126.0 | 0.68 | 40/100 | 85.9 | 0.47 | 8.23 (3.15-21.47) |  |
| No | 598/1762 | 1474.3 | 0.41 | 433/1205 | 1205.9 | 0.36 | 7.10 (3.29-15.34) |  |
| **Viral load ^5^** | | | |  |  |  |  | <0.0001 |
| <5000 Copies/ml | 127/435 | 368.5 | 0.34 | 75/228 | 235.7 | 0.32 | 5.98 (2.87-12.47) |  |
| 5000-30,000 copies/ml | 143/430 | 354.5 | 0.40 | 98/258 | 269.7 | 0.36 | 6.01 (2.55-14.16) |  |
| >30,000 copies/ml | 400/1019 | 830.2 | 0.48 | 265/701 | 671.2 | 0.39 | 8.27 (3.56-19.21) |  |
| **Baseline Clinic Volume^6^** | | |  |  |  |  |  | 0.04 |
| Low Volume | 325/915 | 865.5 | 0.38 | 150/385 | 278.3 | 0.54 | 4.62 (2.46-8.67) |  |
| High volume | 398/1119 | 784.3 | 0.51 | 366/986 | 1053.9 | 0.35 | 8.05 (3.06-21.19) |  |
| **Time from HIV positive to enrollment** | | | |  |  |  |  | 0.60 |
| ≤1 yr, %(n) | 428/1189 | 841.0 | 0.51 | 416/1076 | 947.5 | 0.44 | 6.91 (3.16-15.12) |  |
| 1- ≤3 yr, %(n) | 150/421 | 427.6 | 0.35 | 46/134 | 188.1 | 0.24 | 6.37 (2.64-15.40) |  |
| >3 yr, %(n) | 140/413 | 375.2 | 0.37 | 44/144 | 188.5 | 0.23 | 8.07 (2.89-22.53) |  |
| **Level of clinic** | |  |  |  |  |  |  | 0.0003 |
| Hospital, %(n) | 201/449 | 282.2 | 0.71 | 148/349 | 277.4 | 0.53 | 15.02 (5.35-42.14) |  |
| Clinic with maternity, %(n) | 111/317 | 267.2 | 0.42 | 51/181 | 173.5 | 0.29 | 13.89 (5.14-37.56) |  |
| Clinic without maternity, %(n) | 411/1268 | 1100.4 | 0.37 | 317/841 | 881.3 | 0.36 | 5.80 (2.99-11.22) |  |
| **Access to HIV Treatment supporter** | |  |  |  |  |  |  | 0.77 |
| Yes | 705/1980 | 1610.2 | 0.44 | 503/1339 | 1297.2 | 0.39 | 6.90 (3.12-15.25) |  |
| No | 18/54 | 39.6 | 0.45 | 13/32 | 35.0 | 0.37 | 6.28 (2.13-18.53) |  |
| **Site** |  |  |  |  |  |  |  | 0.52 |
| Bulandzeni Clinic | 17/49 | 19.4 | 0.88 | 49/123 | 154.3 | 0.32 | 49.86 (13.85-179.44) |  |
| Herefords Clinic | 68/168 | 152.5 | 0.45 | 43/73 | 60.8 | 0.71 | 6.39 (3.33-12.25) |  |
| Horo Clinic | 113/313 | 366.3 | 0.3` | 8/44 | 20.7 | 0.39 | 5.35 (2.65-10.78) |  |
| Maguga Clinic | 39/110 | 58.3 | 0.67 | 36/116 | 133.9 | 0.27 | 6.79 (1.85-25.00) |  |
| Malandzela Nazarene Clinic | 14/50 | 29.3 | 0.48 | 24/57 | 56.1 | 0.43 | 79.77 (22.59-281.68) |  |
| Mshingishingini Nazarene Clinic | 7/81 | 17.1 | 0.41 | 32/119 | 157.5 | 0.20 | 29.33 (11.37-75.66) |  |
| Ndvwabangeni Nazarene Clinic | 72/207 | 208.9 | 0.34 | 25/65 | 39.7 | 0.38 | 8.95 (4.14-19.34) |  |
| Pigg's Peak Government Hospital | 201/449 | 282.2 | 0.71 | 148/349 | 277.4 | 0.53 | 14.57 (7.48-28.41) |  |
| Sigangeni Clinic | 54/159 | 171.0 | 0.32 | 21/52 | 27.3 | 0.77 | 51.78 (17.75-151.11) |  |
| Siphocosini Clinic | 82/203 | 188.6 | 0.43 | 27/55 | 28.7 | 0.94 | 28.44 (9.38-86.19) |  |
| Hhukwini Clinic | 35/97 | 97.7 | 0.36 | 12/23 | 13.5 | 0.89 | 77.95 (20.59-295.15) |  |
| Ndzingeni Nazarene Clinic | 1/40 | 16.5 | 0.06 | 26/82 | 86.2 | 0.30 | 15.68 (6.96-35.33) |  |
| Ntfonjeni Clinic | 7/67 | 11.7 | 0.60 | 69/178 | 254.0 | 0.27 | 22.56 (8.40-60.58) |  |
| Peak Nazarene Clinic | 13/41 | 30.2 | 0.43 | 6/35 | 22.1 | 0.27 | 6.01 (2.95-12.23) |  |

^1^ Adjusted for steptime, age (18-24, 25-39, ≥40 years), sex, marital status (Married, Divorced/Widowed, Single), education (Illiterate/Primary, Secondary, High School, Tertiary), CD4 counts at 180 days post ART initiation (<350, 350-500, >500), WHO stage at 180 days post ART initiation (stage 1, stage 2, stage 3 or 4), BMI (<18.5, 18.5-<25,25-<30, ≥30), Screened for TB symptoms (yes, no), viral load (<5‚000, 5‚000-30‚000, >30‚000), treatment support (yes, no), Level of clinic (Hospital, Clinic with maternity, Clinic without maternity), time from HIV tested positive to enrollment (≤1 yr,1- ≤3 yr,>3 yr), clinic volume (Low: < median, High: ≥ median), study enrollment date (2 knots SRCS). If CD4/WHO at 180 days post ART initiation is not available, use CD4/WHO at ART initiation if available, or CD4/WHO if available. Missing data was treated as a separate group for each of the covariates in the models, except for WHO stage at 180 days post ART initiation, CD4 count at 180 days post ART initiation, and BMI , which have too few missing to be a category and the missing was assigned to common category.

^2^ Confidence intervals not adjusted for multiplicity

^3^ Reference group = Standard of Care.

^4^ Robust Wald test accounting for clustering for interaction. Median scores were used for Age, CD4, BMI, and viral load.

^5^ Within 90 days of enrollment and before ART initiation^.^

^6^ First quarter into study
